# Resveratrol downregulates inflammatory pathway activated by lymphotoxin α (TNF-β) in articular chondrocytes: Comparison with TNF-α

**DOI:** 10.1371/journal.pone.0186993

**Published:** 2017-11-02

**Authors:** Constanze Buhrmann, Bastian Popper, Bharat B. Aggarwal, Mehdi Shakibaei

**Affiliations:** 1 Musculoskeletal Research Group and Tumour Biology, Chair of Vegetative Anatomy, Institute of Anatomy, Faculty of Medicine, LMU Munich, Pettenkoferstrasse 11, Munich, Germany; 2 Department of Anatomy and Cell Biology, Biomedical Center, Ludwig-Maximilian-University Munich, Martinsried, Germany; 3 Inflammation Research Centre, San Diego, California, United States of America; University of South Carolina School of Medicine, UNITED STATES

## Abstract

While Lymphotoxin α (TNF-β), a product of lymphocytes, is known to play a pivotal role in inflammatory joint environment, resveratrol has been shown to possess anti-inflammatory and chondroprotective effects *via* activation of the histondeacetylase Sirt1. Whether TNF-β induction of inflammatory pathways in primary human chondrocytes (PCH) can be modulated by resveratrol, was investigated. Monolayer and alginate cultures of PCH were treated with TNF-β, anti-TNF-β, nicotinamide (NAM), antisense oligonucleotides against Sirt1 (Sirt1-ASO) and/or resveratrol and co-cultured with T-lymphocytes. We found that resveratrol suppressed, similar to anti-TNF-β, TNF-β-induced increased adhesiveness in an inflammatory microenvironment of T-lymphocytes and PCH. In contrast, knockdown of Sirt1 by mRNA abolished the inhibitory effects of resveratrol on the TNF-β-induced adhesiveness, suggesting the essential role of this enzyme for resveratrol-mediated anti-inflammatory signaling. Similar results were obtained in PCH stimulated with TNF-α. Sirt1-ASO, NAM or TNF-β, similar to T-lymphocytes induced inflammatory microenvironment by down-regulation of cartilage-specific proteins, Sox9, Ki67 and enhanced NF-κB-regulated gene products involved in inflammatory and degradative processes in cartilage (MMP-9/-13, COX-2, caspase-3), NF-κB activation and its translocation to the nucleus. Moreover, resveratrol reversed the TNF-β-, NAM-, T-lymphocytes-induced up-regulation of various NF-κB-regulated gene products. Down-regulation of Sirt1 by mRNA interference abrogated the effect of resveratrol on TNF-β-induced effects. Ultrastructural and cell viability assay investigations revealed that resveratrol revoked TNF-β-induced dose-dependent degradative/apoptotic morphological changes, cell viability and proliferation in PCH. Taken together, suppression of TNF-β-induced inflammatory microenvironment in PCH by resveratrol/Sirt1 might be a novel therapeutic approach for targeting inflammation during rheumatoid arthritis.

## Introduction

Rheumatoid arthritis (RA) is a chronic, inflammatory, systemic autoimmune disorder characterized by inflammation and degeneration of the synovial joints affecting in particular, the cartilage and the underlying bone [1]. In industrialised countries, RA is the most common inflammatory arthritis affecting 0.5–1% of adults, with 5–50 per 100000 new cases annually and occurring typically in women and elderly people [2]. To date, a well characterized key inflammatory cascade in RA is overproduction and overexpression of TNF-α, enhancing interactions between T- and B-lymphocytes, synovial-like fibroblasts and macrophages, leading to synovial inflammation and joint destruction [3] and further promoting overexpression, release and activity of pro-inflammatory cytokines such TNF-α, vascular endothelial growth factor (VEGF), Interleukin-1β (IL-1β), IL-6, IL-8 and IFN-γ [4, 5]. In response to these pro-inflammatory stimuli, NF-κB-dependently matrix metalloproteinases (MMPs) and cyclooxygenase-2 (COX-2) are produced, progressively degrading the cartilage [6, 7]. Interestingly, it has been previously reported that lymphotoxin α (LT-α) alias TNF-β, another member of the TNF superfamily, may play a critical role in RA [8]. Moreover, TNF-β is the closest homolog to TNF-α showing 35% identity and 50% homology to TNF-α at amino acid sequences and structural similarity in tertiary and quaternary structure indicating similar biological activity [9, 10]. More interestingly, TNF-β is expressed by a variety of cells, including T-cells, B-cells and natural killer (NK) cells [11]. Studies have indicated that TNF-β levels are elevated in the serum and synovial tissue of RA patients and that TNF-β stimulates proliferation and inflammatory cascade signaling in synovial-fibroblasts [8, 12, 13]. Our group could previously show *in vitro* that TNF-β is involved in microenvironment inflammation in chondrocytes similar to IL-1β, resulting in the up-regulation of NF-κB signaling and this could be suppressed by the natural NF-κB inhibitor, curcumin [14].

Disease-modifying anti-rheumatic drugs (DMARDs) such as methotrexate or biological agents such TNF-inhibitors, reduce synovitis and systemic inflammation and improve function [2]. However, they are more often accompanied with severe side effects. In the search for novel effective and safe RA therapies, natural compounds have demonstrated great ability to suppress inflammatory processes through inhibition of pro-inflammatory cytokines. The natural polyphenol resveratrol (3,5,4´-trihydroxy-*trans*-stilbene), found in the skin of red grapes, cranberries, peanuts and root extracts of the weed *Polygonum cuspidatum*, functions microbiologically as a phytoalexin protecting plants against fungal infections and UV radiation [15]. It has been found to possess a wide range of biological and pharmacological activities including anti-oxidative, anti-inflammatory, anti-mutagenic and anti-carcinogenic effects [16]. One of the most important molecular targets of resveratrol is sirtuin-1 (Sirt1), a member of the sirtuin family of nicotinamide adenine dinucleotide (NAD)-dependent deacetylases [17]. The sirtuin family consists of Sirt1-7 and shares a highly conserved NAD^+^-binding catalytic core domain although they exhibit distinct expression patterns, catalytic activities and biological functions [18]. Being able to de-acetylate a wide range of different targets in the cytoplasm and nucleus such as transcription factors p53, Ku70, NF-κB, forkhead proteins, nuclear receptor PPARγ and PGC-α. Indeed, Sirt1 plays an essential role in cell differentiation, cell survival, tumorigenesis, inflammation and metabolism [18–20].

Studies from our laboratory have previously shown that resveratrol possesses anti-inflammatory and chondroprotective effects in human chondrocytes *in vitro* [21–23]. Indeed, resveratrol treatment significantly upregulates Sirt1 expression in normal and osteoarthritic (OA) chondrocytes [24, 25]. Further, in an OA mouse model intra-articular injection of resveratrol significantly prevented the destruction of cartilage by activating Sirt1 [26]. Inflammatory TNFα-induced overexpression of Sirt1 was found to be constitutively upregulated in synovial tissues and cells from patients with RA compared to OA contributing to chronic inflammation and inhibiting apoptosis [27]. Contrary to this, in chondrocytes, TNF-α-induced Sirt1-inactivation correlated with reduced cartilage-specific gene expression [28] and it is widely accepted that Sirt1 exerts positive effects on cartilage by encouraging chondrocyte survival, especially under stress conditions [29].

Several lines of evidence have shown that normal cartilage homeostasis requires enzymatically active Sirt1 protein *in vivo* [30–32]. In fact, decrease of Sirt1 expression was noted during development of OA and reduction of Sirt1 in chondrocytes correlated with enhanced chondrocyte hypertrophy and cartilage matrix loss [33]. Indeed, it has been shown that antagonistic crosstalk between Sirt1 and NF-κB signaling suppresses inflammation, maintains normal bone remodeling, chondrocytes homeostasis and inhibits apoptosis [25, 34–37]. In TNF-α-induced inflammation, Sirt1 deacetylated and inactivated NF-κB, thereby exerting its anti-inflammatory effect in chondrocytes [38]. Further, reports show that Sirt1 plays a protective role by suppressing IL-1β- or TNF-β-induced expressions of cartilage-degrading enzymes by modulation of the NF-κB pathway and NF-κB-dependent-gene-end-products [25, 39]. However, the role of resveratrol induced Sirt1-activation during TNF-β-induced inflammatory environment in RA/OA is widely unknown and needs to be further elucidated.

The purpose of this study was therefore, to examine the effect of resveratrol-induced Sirt1 signalling on suppressing inflammatory pathways activated by TNF-β in primary human chondrocytes as potential novel therapeutic approach for the treatment of inflammatory joint diseases such as RA/OA.

## Materials and methods

### Antibodies and cytokines

Anti-active caspase-3, -MMP-9, -MMP-13, -TNF-α and TNF-α were purchased from R&D Systems, Inc., (Heidelberg, Germany). Antibodies to phospho-specific p65 (NF-κB)/(Ser536) were purchased from Cell Technology (Beverly, MA, USA). Antibodies to collagen type II and alkaline phosphatase-linked sheep anti-mouse and sheep anti-rabbit secondary antibodies for immunoblotting were purchased from Millipore (Schwalbach, Germany). Anti-TNF-β was obtained from eBioscience (Frankfurt, Germany). Anti-Ki67 and secondary antibodies used for fluorescence labelling were purchased from Dianova (Hamburg, Germany). Anti-cyclooxygenase-2 was purchased from Cayman Chemical (Ann Arbor, MI, USA). Monoclonal anti-β-Actin was obtained from Sigma-Aldrich (Munich, Germany). Sox9 antibody was purchased from Acris Antibodies GmbH (Hiddenhausen, Germany). Polyclonal antibody against Sirt1 was purchased from Abcam PLC (Cambridge, UK).TNF-β and polyclonal rabbit anti-TNF-β were obtained as described [40]. Bacteria-derived recombinant human TNF-α and LT-α (TNF-β), both purified to homogeneity with a specific activity of 50 million units/mg, were kindly gift from Genentech, Inc. (South San Francisco, CA).

### Growth media and chemicals

Cell culture growth medium was prepared as previously described [25]. Dulbecco’s modified Eagle’s medium/Ham’s F-12 (1:1), 10% fetal bovine serum (FBS), 0,5% amphotericin B solution, 1% penicillin/streptomycin solution (10,000 IU/10,000 IU), 75 μg/ml ascorbic acid, 1% essential amino acids and 1% glutamine was obtained from Seromed (Munich, Germany). Epon was purchased from Plano (Marburg, Germany). Alginate, Nicotinamide (NAM) and resveratrol with purity greater than 98% were purchased from Sigma (Munich, Germany). A 100mM stock solution of resveratrol (molecular weight 228.2) was prepared in ethanol and further diluted in cell culture medium to prepare working concentrations. The maximum final content of ethanol in cultures was less than 0.1%. This concentration was also used as a control. The stock solution was stored at -20°C.

### Chondrocyte and T-lymphocyte culture

Primary human chondrocytes (HCHON, catalog no. 121 0211) were purchased from Provitro (Berlin, Germany). Cartilage samples of knee joint used for chondrocyte isolation were derived from human patient (50 years old, female undergoing total knee replacement), who provided her full informed consent. Participant gave written informed consent. This study was carried out in accordance with the declaration of Helsinki and local ethics committee approval was provided by the Charité-University Medical School Berlin, Germany. Briefly, during monolayer expansion, chondrocytes were seeded at a density of 300,000 cells/T75 cell culture flask and grown until 70% confluence was reached in cell culture growth medium (10% FBS). Chondrocytes were passaged up to two times, and passages 2 and 3 were used in the experiments. Chondrocytes were washed three times with serum-starved medium (3% FBS) and further incubated for 30 minutes with the same medium before initiation of experiments. All experiments were performed with serum-starved medium. A human T-lymphocyte cell line (Jurkat cells) [41] was cultured in suspension with whole-cell culture growth medium as described above.

### Antisense and Lipofectin-mediated transfection in chondrocytes

Transient transfection of primary human chondrocytes was performed as previously described in detail [25, 42]. The Sirt1 antisense sequences used in these experiments were designed using a computational neural network mode [43]. Briefly, Phosphorothioate-specific oligonucleotides (21-mer) in antisense (ASO) (sequence 5´-GTATTCCACATGAAACAGACA-3´) corresponding to Sirt1 mRNA and control 21-mer sense oligonucleotides (SO) (sequence 5´-TGTCTGTTTCATGTGGAATAC-3´) were synthesized by eurofins (mwg/operon, Ebersberg, Germany). All ASOs and SOs were phosphorothioate-modified to protect them from cell nucleases. Transfection was carried out by incubating the primary chondrocytes in monolayer culture for 24h with 0.5μM ASO against Sirt1 or SO control and 10μl/ml Lipofectin transfection reagent (Invitrogen, Carlsbad, CA, USA) in serum-starved medium before starting the respective experiments. All transfection experiments were carried out on 50% confluent monolayer cultures.

### Adhesion assay interaction of chondrocytes with T-lymphocytes

For investigation of interaction between chondrocytes with T-lymphocytes an adhesion assay was performed as previously described [14]. Briefly, primary human chondrocytes were grown to subconfluence in monolayer culture and either left untreated alone or co-cultured with T-lymphocytes (Jurkat cells) (1×10^6^/ml), or were treated with 10ng/ml TNF-β, or 10ng/ml TNF-β and 5μl/ml anti-TNF-β, or 10ng/ml TNF-α, or 10ng/ml TNF-α and 5μl/ml anti-TNF-α and then co-cultured with T-lymphocytes for 8h. Additionally, in another set of experiments, primary human chondrocytes were either co-cultured with T-lymphocytes and/or with 5μM resveratrol and/or 10ng/ml TNF-β and/or 10ng/ml TNF-α for 8h, or were transfected with Sirt1-ASO or Sirt1-SO in the presence of Lipofectin (10μl/ml) for 24h prior to co-treatment with T-lymphocytes and 5μM resveratrol and 10ng/ml TNF-β or 10ng/ml TNF-α for 8h. After 8h of co-culture, non-adherent T cells were carefully removed by gentle washing once with Hank´s balanced salt solution (Biochrom, Germany) and photographed under a light microscope (Zeiss, Jena, Germany). The number of adhered T-lymphocyte colonies on chondrocytes was determined by scoring 10 different microscopic fields. This experiment was repeated three times independently and statistical analysis was done to obtain the final values.

### Immunofluorescence analysis

The technique was performed as previously described in detail [42]. Briefly, primary human chondrocytes were either left untreated or treated with 5μM resveratrol, or with 10ng/ml TNF-β, or co-cultured with T-lymphocytes (1×10^6^/ml) alone for 12h. Additionally, primary human chondrocytes were co-cultured with T-lymphocytes and either treated with 10ng/ml TNF-β, or with 5μM resveratrol, or with 10ng/ml TNF-β and 5μM resveratrol for 12h. Cells were washed in Hanks’ solution (PBS) before methanol fixation and permeabilization of the cells for 10 min at ambient temperature (AT). This was followed by rinsing 3 times with a mixture of protease-free bovine serum albumin (BSA) and PBS for 10min at AT and then incubated with primary antibodies (Sirt1, NF-κB, 1:80 in PBS/BSA) in a humid chamber overnight at 4°C. Cultures were washed three times with PBS/BSA before incubation with rhodamine-red conjugated secondary antibody (diluted 1:80 in PBS/BSA) for 1.5h at AT and finally rinsed again three times with PBS. Counter-staining was performed with 4′,6-diamidino-2-phenylindole (DAPI; Sigma-Aldrich Chemie) to visualize cell nuclei for 10 min. Cells were finally washed once with aqua dest. and covered with fluoromount mountant (Sigma). Samples were evaluated under a fluorescence microscope (Leica Microsystems, Wetzlar, Germany). Quantification of positively stained cells was performed by scoring 100 cells from 10 different microscopic fields.

### Transmission electron microscopy (monolayer- and alginate cultures)

Serum-starved primary human chondrocytes were either left untreated, treated with various concentrations of TNF-β (1, 5, 10 or 20ng/ml) for 1h, or co-treated with resveratrol 5μM and various concentrations of TNF-β (0, 1, 5, 10 or 20ng/ml) for 1h in monolayer cultures. In a second approach, monolayer cultured chondrocytes (1x10^6^/ml) were transferred to alginate cultures, treated as described above and cultured under similar conditions with serum-starved medium for 10 days to examine the effects of TNF-β and/or resveratrol on chondrocytes differentiation potential in a three-dimensional environment. Three-dimensional alginate cultures were prepared as previously described [44, 45]. Briefly, the pellet of chondrocytes (1×10^6^/ml) was resuspended in alginate (2% in 0.15M NaCl, stirring for 1–2 h) and slowly added dropwise into a solution containing 100mM CaCl_2_ at ambient temperature (AT). The alginate beads polymerized in the presence of CaCl_2_ after 10 min. Subsequently, the CaCl_2_ solution was removed and the alginate beads rinsed three times with 0.15M NaCl solution and twice with serum-starved medium (3% FBS).The medium was changed every 3 days. The cultures were grown in an incubator at 37°C with 5% CO_2_ in air. These experiments were performed in triplicate, and the results are provided as mean values from three independent experiments. A detailed description of the culture technique used for transmission electron microscopy has been published [44, 45]. After fixation and post fixation in 1% tannic acid and 1% O_s_O_4_ solution, cells from monolayer or alginate cultures were rinsed and dehydrated in ascending alcohol series. They were embedded in Epon, cut on a Reichert Ultracut (Leica, Wetzlar, Germany) followed by contrasting with 2% uranyl acetate/lead citrate. For inspection a transmission electron microscope (EM 10 Zeiss, Institute of Pharmacology, Berlin, Germany) or a Jeol 1200 EXII, Akishima Tokyo, Japan (Department of Anatomy and Cell Biology, Martinsried, Germany) was used.

### Quantification of apoptotic cell death

To quantify apoptosis and cells with mitochondrial changes, ultrathin sections of the samples were prepared and evaluated with a transmission electron microscope. The number of cells exhibiting typical morphological features of apoptotic cell death was determined by scoring 100 cells from 25 different microscopic fields per culture. The values were initially subjected to one-way ANOVA and then later compared among groups using unpaired Student’s t-test, followed by a post-hoc test to compare the parameters of each group.

### MTT assay from alginate bead culture

To evaluate cell viability of chondrocytes in alginate bead culture, cells were retrieved from alginate, as previously described in detail [44] and a MTT assay (3-(4,5-dimethylthiazol-2-yl)-2,5-diphenyltetrazolium bromide) was performed. Briefly, alginate beads were left untreated or treated with various concentrations of TNF-β (0.1, 1, 5, 10 or 20ng/ml) or were co-stimulated with resveratrol 5μM and various concentrations of TNF-β (0, 0.1, 1, 5, 10 or 20ng/ml) for 14 days. To release the cells from the alginate, alginate beads were washed two times with Hanks Salt Solution and dissolved in 55mM sodium citrate solution for 20–30 min. Subsequently, in triplicate, 100 μl of cell suspension was distributed to a 96-well-plate, to each well were immediately added 10 μl MTT solution (5mg/ml) and the plate was incubated for 4h at 37°C. Finally, 100μl of the MTT solubilisation solution (10% Triton x-100/acidic isopropanol) was added per well, and the cells incubated overnight at 37°C. Metabolically active cells were evaluated through measuring the Optical Density at 550nm (OD550) using revelation 96-well multiscanner plate ELISA reader (Bio-Rad Laboratories Inc. Munich, Germany). The results obtained were calculated and were represented as percentage of survival relative to controls. The experiments were repeated three times independently and statistical analysis was done to obtain the final values.

### Western blot analysis

Primary human chondrocytes in monolayer cultures were either left untreated or treated with 5μM resveratrol, or 10ng/ml TNF-β, or 5μM resveratrol and 10ng/ml TNF-β or co-cultured with T-lymphocytes (1×10^6^/ml) alone for 4h, or co-cultured with T-lymphocytes and additionally treated with 10ng/ml TNF-β, or 5μM resveratrol or with 10ng/ml TNF-β and 5μM resveratrol for 4h. In a second approach, serum-starved monolayer cultured primary human chondrocytes were either left untreated or treated with 5μM resveratrol, or 10ng/ml TNF-β, or 10mM NAM, or were co-treated with 5μM resveratrol and 10ng/ml TNF-β or 5μM resveratrol and 10mM NAM for 4h. Additionally, chondrocytes were transfected with 0.5μM Sirt1-SO or Sirt1-ASO in the presence of Lipofectin (10μl/ml) for 24h and either left untreated or co-treated with resveratrol (5μM) for 4h. In another set of experiments, serum-starved chondrocytes in alginate beads were left untreated or treated with various concentrations of TNF-β (0.1, 1, 10ng/ml), or were co-stimulated with 5μM resveratrol and various concentrations of TNF-β (0.1, 1, 10ng/ml) for 14 days. The whole-cell extracts were prepared for immunoblotting as previously described [25, 44, 46]. Whole cell lysate proteins were extracted from cultures on ice for 30 min. with lysis buffer (50mM Tris-HCl, pH 7.2, 150mM NaCl, 1% (v/v) Triton X-100, 1mM sodium orthovanadate, 50mM sodium pyrophosphate, 100mM sodium fluoride, 0.01% (v/v) aprotinin, 4 mg/ml of pepstatin A, 10mg/ml of leupeptin, 1mM phenyl methyl sulfonylfluoride, PMSF), total protein concentration was measured with the bicinchonic acid assay system (Uptima, Monlucon, France) using bovine serum albumin as a standard, and Subsequently, samples were reduced with 2-mercaptoethanol and equal quantities of protein (500ng/lane), separated under reducing conditions by SDS-PAGE. After transfering onto nitrocellulose membranes using a transblot apparatus (Bio-Rad) and pre-incubation in blocking buffer (5% skimmed milk powder in PBS, 0.1% Tween 20) for 2h, membranes were incubated with primary antibodies at 4°C overnight, washed three times with blocking buffer, and then further incubated with alkaline phosphatase-conjugated secondary antibodies for 2h at AT. After further washing in 0.1M Tris, pH 9.5, containing 0.05M MgCl_2_ and 0.1M NaCl, specific antigen-antibody complexes were detected using nitro blue tetrazolium and 5-bromo-4-chloro-3-indoylphosphate (p-toluidine salt; Pierce). Total protein amount was defined according to the bicinchoninic acid system (Pierce, Rockford, IL, USA) using bovine serum albumin as a standard. Specific binding was quantified by densitometry using ‘‘quantity one” (Bio-Rad Laboratories Inc. CA, USA). Specific β-actin antibody was used for the internal control to normalize the sample amounts.

### Statistical analysis

Experiments were performed three times as individual experiments with three replicates. For statistical analysis, a Wilcoxon-Mann-Whitney test was applied. Score values for image quality and presence of artifacts were compared for each sequence. A *p* value of <0.05 was considered to establish statistically significant differences.

## Results

The aim of the present study was to examine whether resveratrol could inhibit the inflammatory role of TNF-β in an inflammatory microenvironment of primary human chondrocytes and T-lymphocytes *in vitro*. To determine this, the mechanism by which resveratrol manifests its effect was investigated against inflammatory pathways activated by TNF-β in chondrocytes in monolayer and alginate cultures *in vitro*. The concentration of resveratrol or ethanol applied in our study and the time of exposure had no suppressive effect on chondrocyte cell viability.

### Knock-down of Sirt1 modulates resveratrol-induced inhibition of TNF-β- or TNF-α-mediated adhesiveness of T-lymphocytes to chondrocytes *in vitro*

**Fig 1:**
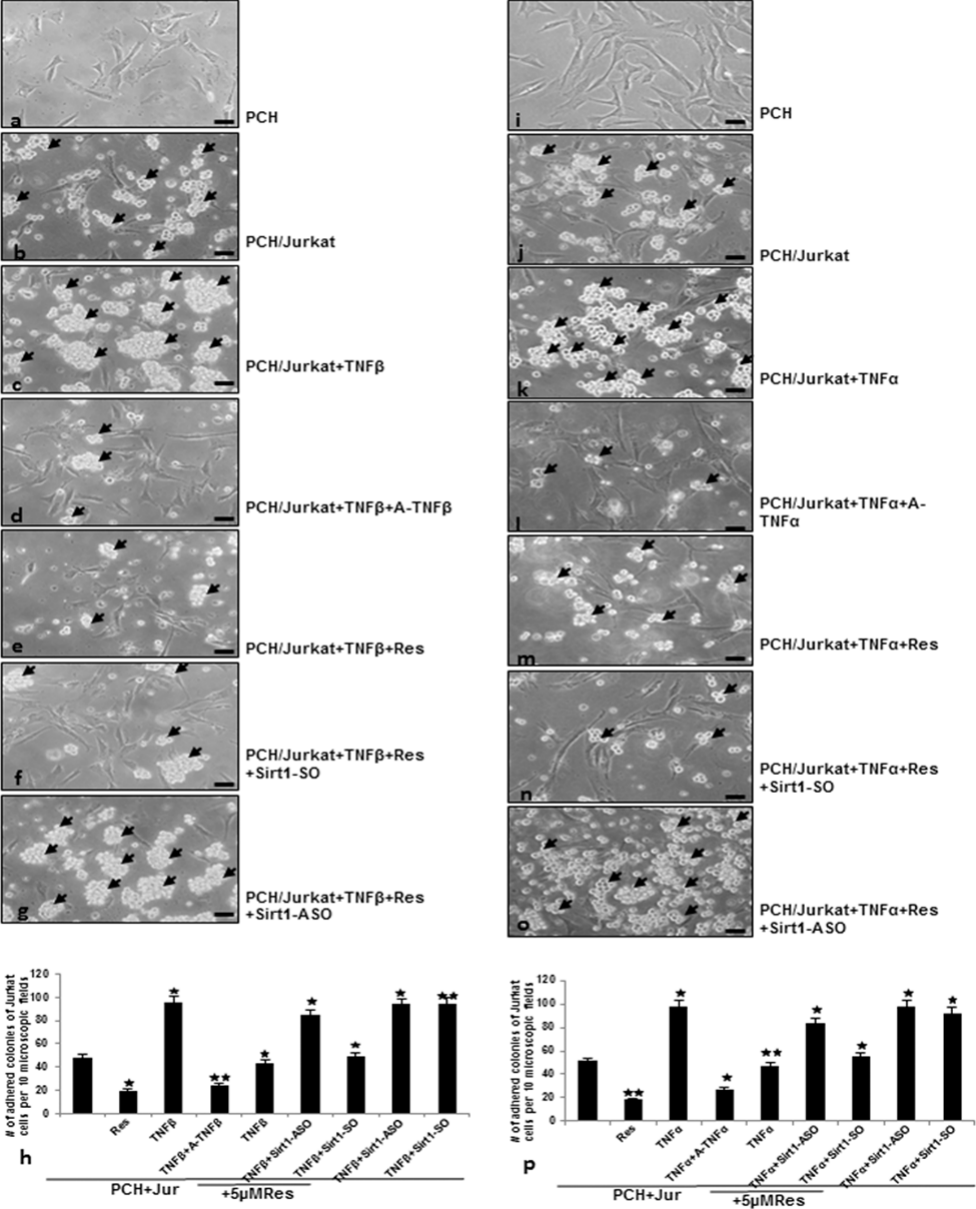
Effects of resveratrol and ASO against Sirt1 or TNF-β- or to TNF-α-mediated adhesiveness of T-lymphocytes to chondrocytes. Serum-starved human primary chondrocytes (PCH) were cultured to sub-confluence in monolayer culture and were either left untreated or co-cultured with T-lymphocytes and treated with TNF-β (a-g) or TNF-α (i-o) as described in Material and Methods. After being washed with phosphate-buffered saline, adhesion of T-lymphocytes (arrows) to chondrocytes was evaluated by light microscopy. Original magnification, x400; bar, 30nm. The number of adhered colonies of T-lymphocytes to chondrocytes was estimated and quantified by counting 10 microscopic fields per culture (h and p).

It has been shown that inflammation in RA is mediated also through recruitment of lymphocytes to the site of inflammation [47] and our group could previously demonstrate that TNF-β treatment of chondrocytes with increased dosages of TNF-β resulted in an almost 2fold increase in the adherence of T-lymphocytes [14], highlighting a novel role for TNF-β in supporting an inflammatory milieu and stimulating ongoing inflammation in chondrocytes. To investigate further the role of TNF-β signaling in attracting T-lymphocytes and inducing an inflammatory microenvironment, we performed an adhesion assay of chondrocytes with T-lymphocytes. Serum starved chondrocytes were grown to sub-confluence in monolayer culture (Fig 1) and either cultured alone or co-cultured with T-lymphocytes or co-cultured with T-lymphocytes and treated as described in Material and Methods. Stimulation of co-cultures with TNF-β or TNF-α resulted in 2 to 2.5 fold increase in the adherence of T-lymphocytes to chondrocytes compared to control, underlining the role of these cytokines in stimulating and supporting an inflammatory microenvironment (Fig 1B, 1C, 1J and 1K). In contrast to this, treatment of co-cultures with TNF-β and anti-TNF-β similar to TNF-α and anti-TNF-α, or resveratrol with either TNF-β or TNF-α markedly suppressed T-lymphocyte adherence to chondrocytes (Fig 1D, 1E, 1L and 1M). As resveratrol has been reported to be a specific potent activator of histone deacetylase Sirt1 [48], we additionally investigated whether resveratrol-suppression of TNF-β-induced inflammatory adherence of T-lymphocytes to chondrocytes is mediated *via* a Sirt1 dependent pathway. Prior to co-culture with T-lymphocytes and co-treatment with resveratrol, TNF-β or TNF-α, chondrocytes were transfected with Sirt1-ASO or Sirt1-SO, as described in Materials and Methods (Fig 1F, 1G, 1N and 1O). In TNF-β- or TNF-α-stimulated co-cultures transfected with control Sirt1-SO, T-lymphocyte adherence to chondrocytes was suppressed by resveratrol treatment. In contrast, knockdown of Sirt1 with ASO against Sirt1 clearly increased TNF-β- or TNF-α-induced adherence of T-lymphocytes in spite of resveratrol treatment. These results indicate clearly that resveratrol-induced suppression of inflammatory microenvironment in chondrocytes by TNF-β is mediated, at least in part *via* activation of a Sirt1 dependent pathway. Statistical evaluation of the Jurkat cell colonies adherent to chondrocytes was estimated and quantified by counting 100 colonies from 25 microscopic fields. As shown in Fig 1H and 1P, this confirmed the results from Fig 1A–1G and 1I–1O.

### TNF-β- similar to T-lymphocytes-induced NF-κB-signalling in chondrocytes is suppressed by resveratrol-activated Sirt1 up-regulation

**Fig 2:**
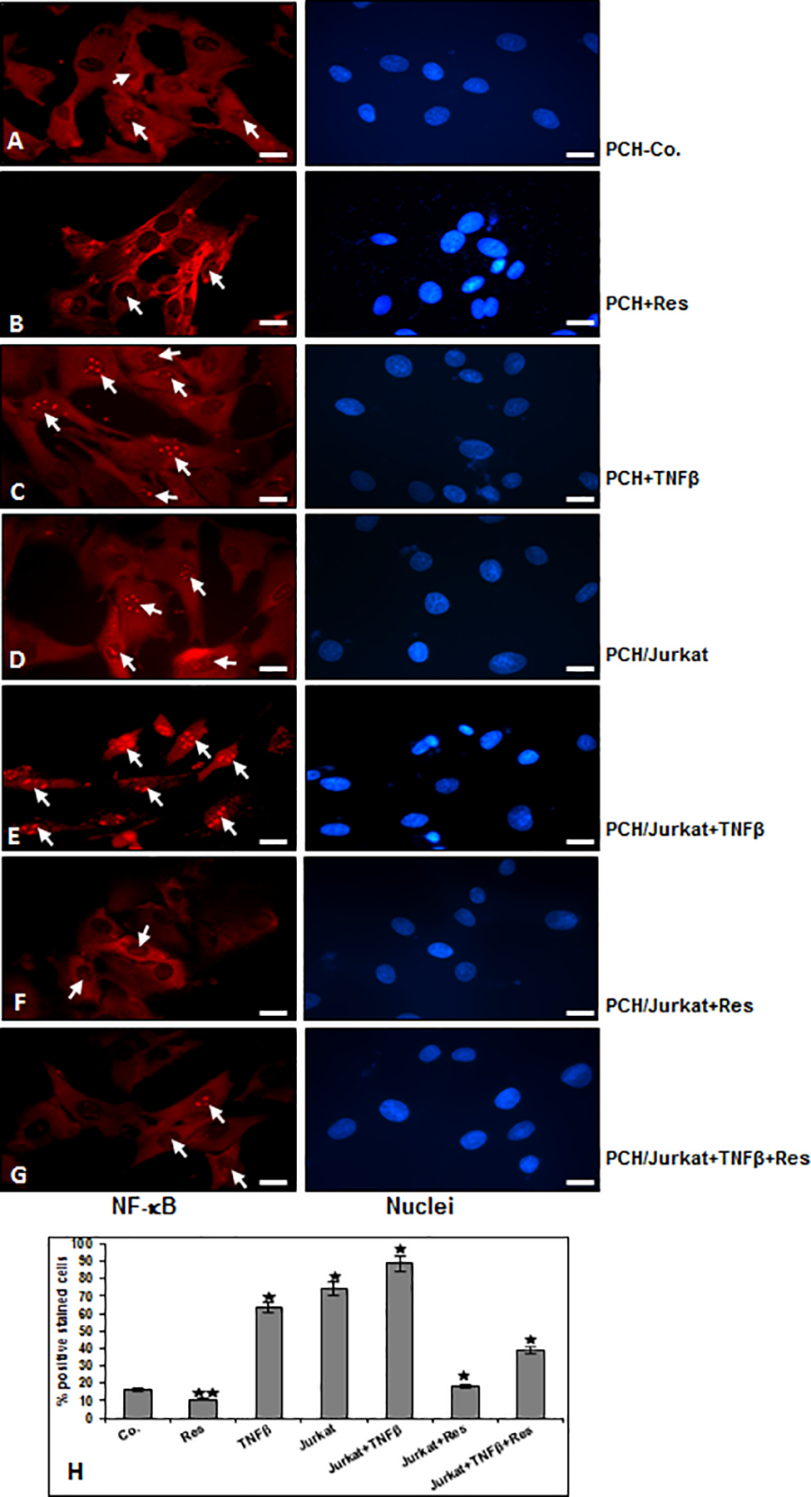
Immunohistochemical analysis of p65 localization after treatment with TNF-β and/or T-lymphocytes and/or resveratrol as revealed by immunofluorescence microscopy in chondrocytes. A-G: NF-κB-p65 localization in human chondrocytes after treatment with resveratrol, TNF-β and/or co-culture with T-lymphocytes as described in Material and Methods. For immunolabeling, cells were incubated with primary antibodies against NF-κB-p65, followed by incubation with rhodamine-coupled secondary antibodies and counterstaining with DAPI to visualize cell nuclei. Original magnification, x400; bar, 30nm. To quantify the chondrocytes with nuclear-positive translocation p65 (H), the labeled cultures were examined by counting 100 cells from 10 microscopic fields. The results are provided as the mean values with S.D. from three independent experiments. Values were compared with the control, and statistically significant values with p<0.05 are designated by (*), and p<0.01 is designated by (**).

To examine whether the observed enhanced adhesiveness of T-lymphocytes to chondrocytes by TNF-β or T-lymphocytes is correlated/associated with up-regulation of inflammatory signalling in chondrocytes, we performed immunocytochemical analysis for phosphorylated-NF-κB (Fig 2). It has been reported that translocation of NF-κB to the nucleus is necessary for the regulation of gene expression by NF-κB and the translocation of activated NF-κB is preceded by phosphorylation of the p65 subunit of NF-κB [49, 50]. Serum starved chondrocytes were cultured on glass plates in monolayer and were either left untreated, treated with resveratrol, TNF-β or co-cultured with T-lymphocytes and treated as described in Material and Methods. In untreated chondrocytes or treated with resveratrol, inactivated NF-κB was mainly found in the cytoplasm (Fig 2A and 2B). In contrast, treatment with TNF-β alone, co-culture with T-lymphocytes or co-culture with T-lymphocytes and TNF-β markedly induced NF-κB activation in chondrocytes through enhanced nuclear translocation of p65 (Fig 2C–2E). Treatment with resveratrol alone (Fig 2B), or co-treatment with TNF-β (not shown) or in combination with T-lymphocytes alone (Fig 2F) or T-lymphocytes in combination with TNF-β (Fig 2G) strongly suppressed NF-κB activation as shown by significantly fewer NF-κB-nuclear positive stained cells. These results indicate that resveratrol-induced suppression of TNF-β- similar to T-lymphocytes-enhanced inflammatory signaling in chondrocytes is mediated also through inhibition of NF-κB activation. Quantification of NF-κB-nuclear positively stained cells confirmed the immunofluorescence results (Fig 2H).

**Fig 3:**
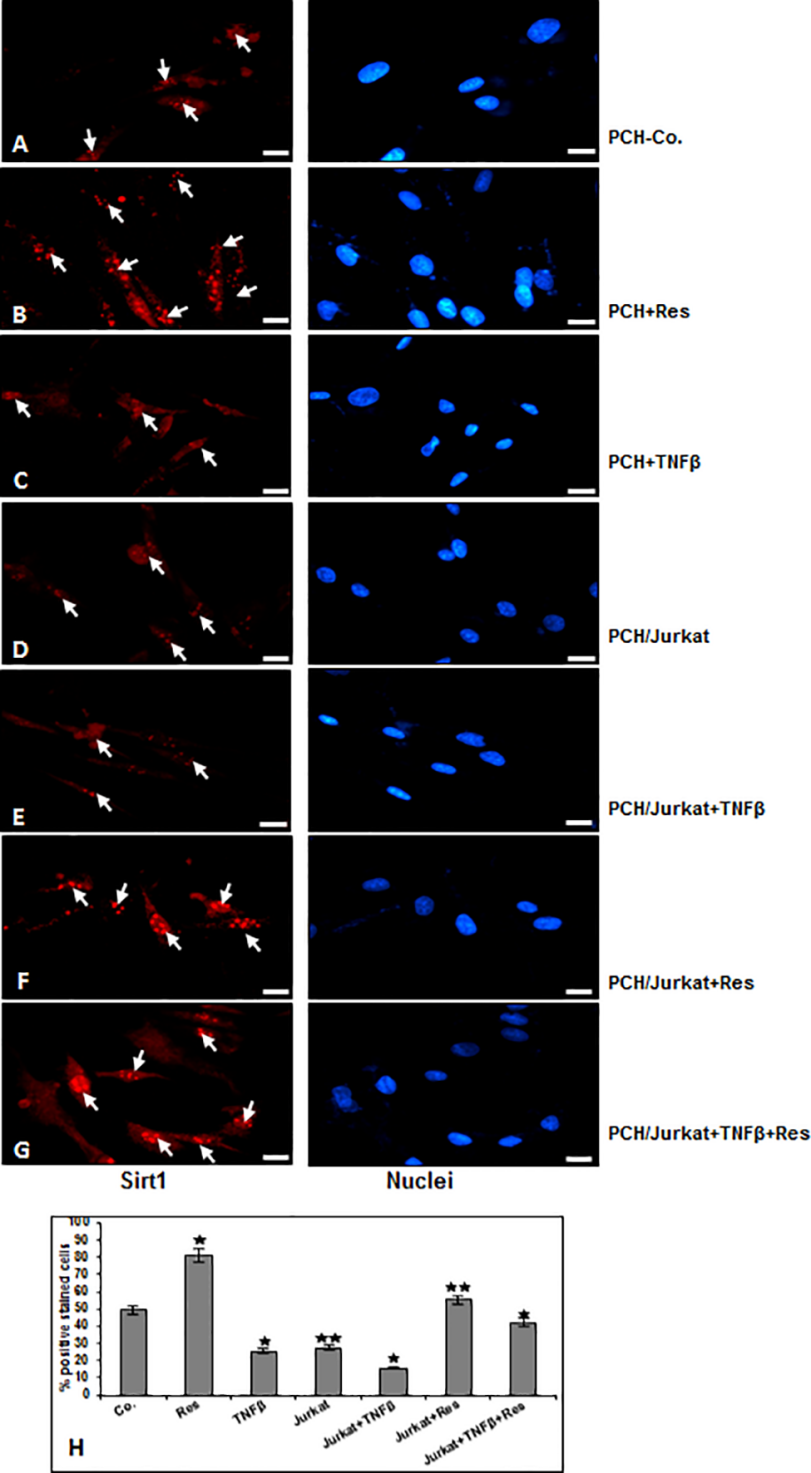
Effect of resveratrol and/or TNF-β and/or T-lymphocytes on the expression of Sirt1 revealed by immunofluorescence in chondrocytes. A-G: Sirt1 expression in human chondrocytes after treatment with resveratrol, TNF-β and/or co-culture with T-lymphocytes as described in Material and Methods. Immunolabeling was performed with primary antibodies against Sirt1, followed by incubation with rhodamine-coupled secondary antibodies and counterstaining with DAPI to visualize cell nuclei. Original magnification, x400; bar, 30nm. Quantification of cells with positive nuclear labeling (H) was examined by counting 100 cells from 10 microscopic fields. The results are provided as the mean values with S.D. from three independent experiments. Values were compared with the control, and statistically significant values with p<0.05 are designated by (*), and p<0.01 is designated by (**).

We could previously show that there is a causal relationship between Sirt1 and NF-κB signaling pathway during chondrogenesis [25]. Therefore, next we examined whether the observed suppression of NF-κB inflammatory pathway by resveratrol in chondrocytes by TNF-β and/or T-lymphocyte is mediated *via* Sirt1 activation. Serum starved chondrocytes were treated as described above and submitted to immunofluorescent labelling with anti-Sirt1 (Fig 3). The immunofluorescence analysis revealed that treatment of chondrocytes with TNF-β or co-culture with T-lymphocytes or co-culture with T-lymphocytes and TNF-β strongly suppressed Sirt1 nuclear localization compared to control (Fig 3C–3E). In contrast to this, treatment with resveratrol alone (Fig 3B) or in combination with T-lymphocytes alone or with T-lymphocytes in combination with TNF-β (Fig 3F and 3G) significantly upregulated Sirt1 nuclear localization as confirmed by quantification of positively stained cells (Fig 3H). Taken together, these findings indicate that resveratrol-activated Sirt1 suppressed inflammatory-induced signaling by TNF-β similar to T-lymphocytes in chondrocytes, highlighting the crucial role of Sirt1 in inhibiting TNF-β-induced inflammatory environment by the NF-κB pathway.

### Resveratrol suppresses TNF-β-induced degradation and apoptosis in chondrocytes in monolayer and alginate cultures

**Fig 4:**
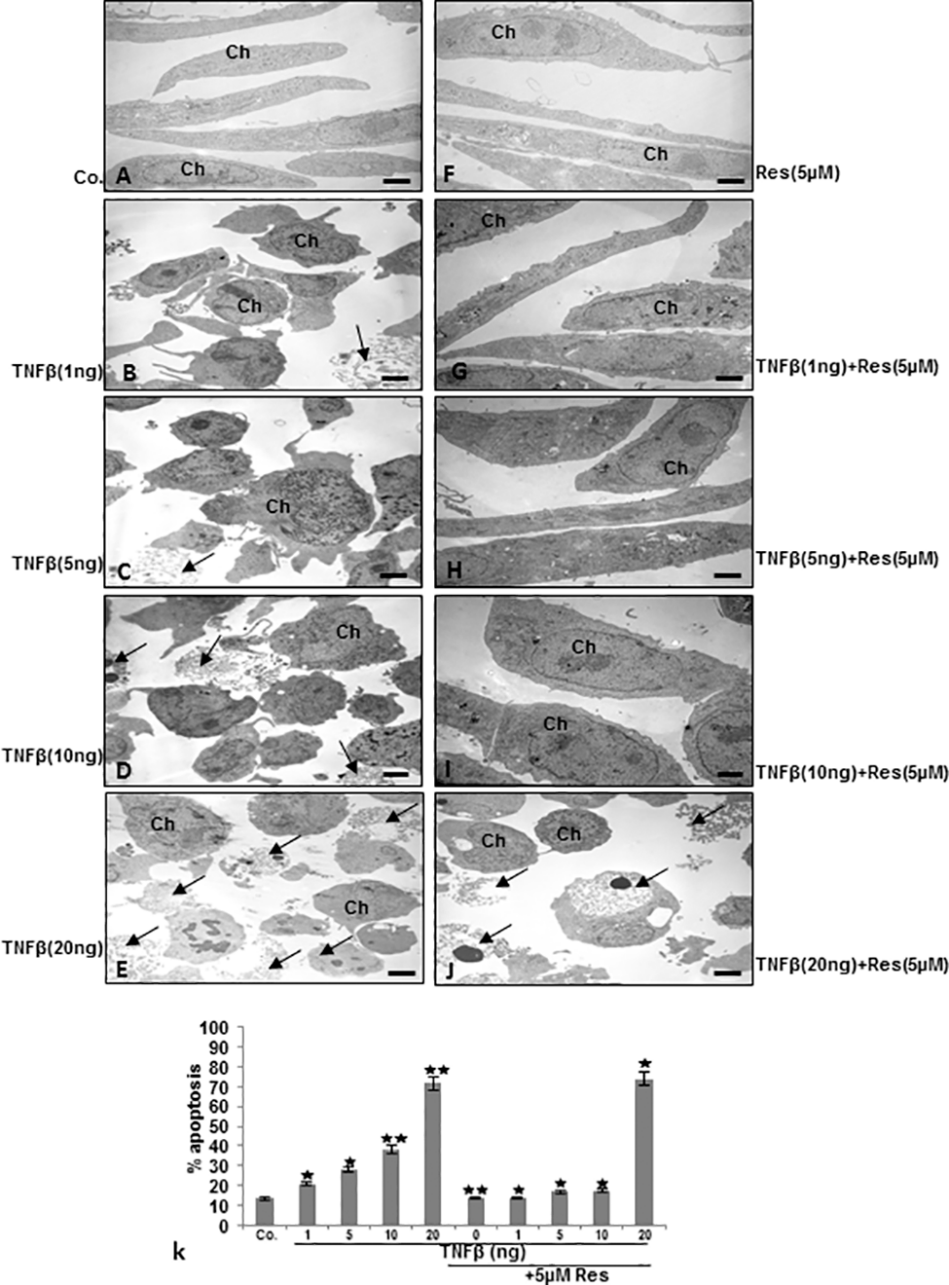
Electron microscopic evaluation on cellular degeneration and apoptosis in chondrocytes in monolayer cultures after treatment with resveratrol and/or TNF-β. A-J: Serum-starved human primary chondrocytes (Ch) were cultured to sub-confluence in monolayer culture and were either left untreated or treated with different concentration of TNF-β (1, 5, 10 and 20ng/ml) and/or co-treated with resveratrol and evaluated with a transmission electron microscope. Untreated control (Co.), chondrocytes containing mitochondria, rough endoplasmic reticulum and many other cell organelles (A). In contrast, treatment of chondrocytes with TNF-β resulted in degenerative changes of the cells. Cells became rounded and the nucleus contained more condensed chromatin, multiple vacuoles, swelling of rough endoplasmic reticulum, and clustering of swollen mitochondria was visible. Higher concentration of TNF-β (10, 20ng/ml) led to the formation of apoptotic bodies and cell lysis (arrows). A-J: x5000; bar = 1μM. k: To quantify cellular degradation and apoptosis in chondrocytes monolayer cultures, 100 cells from 25 microscopic fields were counted and results presented are mean values with standard deviations from three independent experiments. Values were compared with the control and statistically significant values with p<0.05 were designated by (*) and p<0.01 were designated by (**).

**Fig 5:**
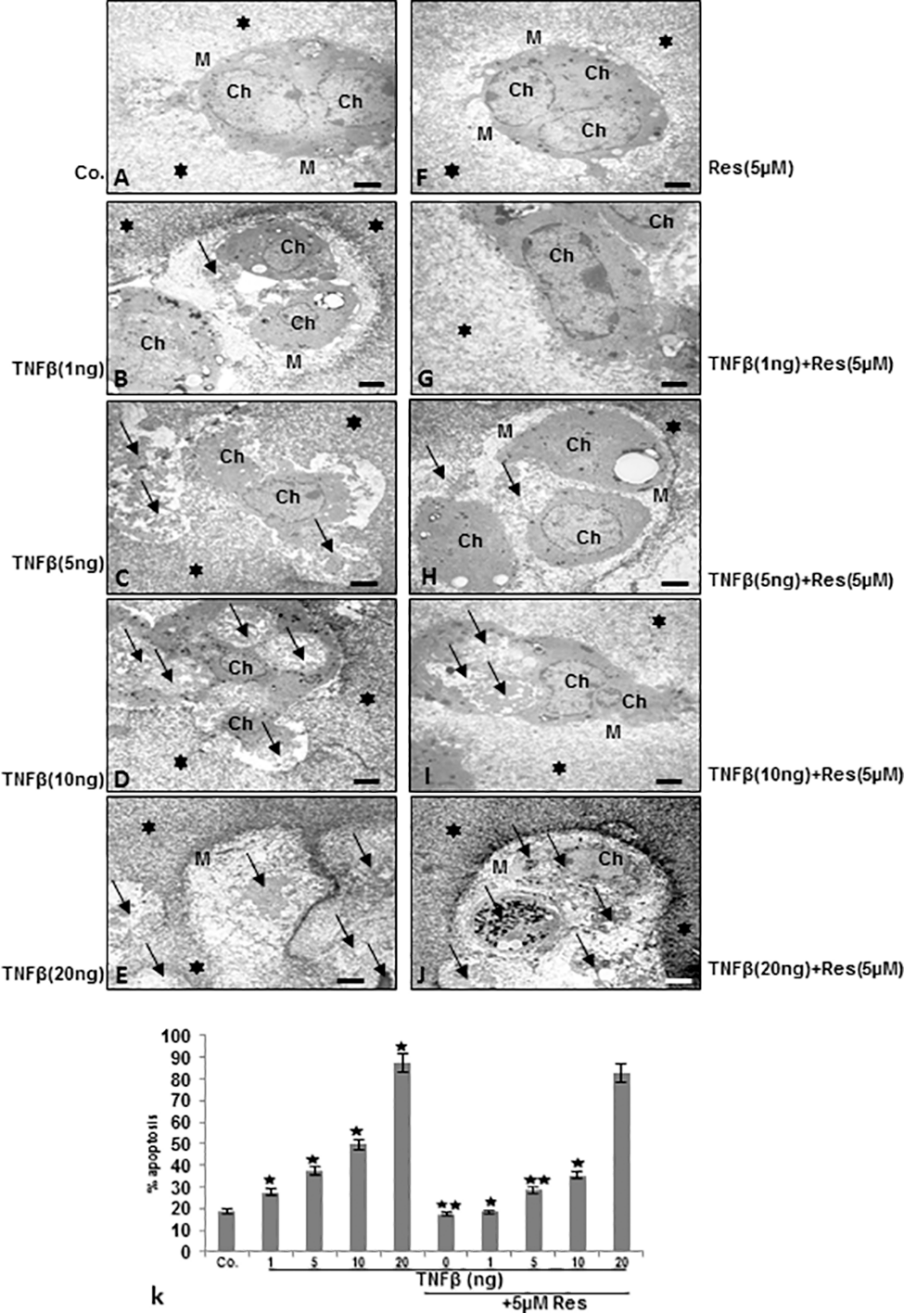
Effect of resveratrol on TNF-β-induced cellular degeneration and apoptosis in chondrocytes in 3-D alginate cultures. A-J: Serum-starved human primary chondrocytes (Ch) were cultured in alginate beads (*) and were either left untreated (Co.) or were treated with different concentrations of TNF-β (1, 5, 10 and 20ng/ml) or resveratrol (5μM) or a combination of resveratrol (5μM) and TNF-β (1, 5, 10 and 20ng/ml) for 14 days. Magnification: x5000, bar = 1μM. k: morphological features of apoptotic cell death (arrows) were quantified by counting 100 in cells from 25 different microscopic fields and results presented are mean values with standard deviations from three independent experiments. Significant values were compared with the control and statistically significant values with p<0.05 were designated by (*) and p<0.01 were designated by (**).

For up-keeping of chondrogenic potential, cell-cell and cell-matrix interaction between chondrocytes and their surrounding matrix are essential [51]. Therefore, to examine whether the observed TNF-β-induced inflammatory signaling pathways in chondrocytes are associated with degenerative cell morphology changes and loss of cell-cell and cell-matrix interaction, ultrastructural electron microscopic investigations in monolayer and 3-D microenvironment alginate culture were performed (Figs 4 and 5). For monolayer investigations, chondrocytes were either left untreated or treated with various concentrations of TNF-β as described in Material and Methods. Untreated or resveratrol treated chondrocytes in monolayer cultures showed typical fibroblast-like shape with small cytoplasmic processes, a large, mostly euchromatic nucleus with nucleoli and a well-structured cytoplasm (Fig 4A and 4F). Treatment with TNF-β revealed substantial degenerative changes in chondrocytes morphology, typical microvilli-like processes disappeared, cell shapes became rounded, multiple vacuoles appeared as well as swollen and degenerated cell organelles (especially mitochondria, rough ER) and cell nuclei containing more condensed heterochromatin could be observed. A clear dose-dependent increase of TNF-β-induced morphological signs of degeneration and degradation in chondrocytes was observed. The dosage of 20ng/ml TNF-β caused almost completely cell degradation and apoptosis (Fig 4B–4E). In contrast to this, co-treatment with resveratrol, resulted in cells with fewer apoptotic features in cultures treated with up to 10ng/ml TNF-β, demonstrating a resveratrol-induced protective effect against TNF-β-induced cellular degeneration and apoptosis in chondrocytes (Fig 4G–4J). To confirm the morphological findings and quantify them, cells displaying severe mitochondrial changes or apoptosis were counted among 100 cells from 25 microscopic fields (Fig 4k). In monolayer culture, TNF-β effectively induced apoptosis in a dose-dependent manner with 38% apoptotic cells at 10ng/ml, 28% at 5ng/ml, 21% at 1ng/ml compared to 13% in untreated or 12% in resveratrol treated chondrocytes. Apoptosis in resveratrol and TNF-β co-treated cultures was significantly lower with 18% at 10ng/ml, 18% at 5ng/ml and 13% at 1ng/ml TNF-β. Dose of 20ng/ml TNF-β alone or in combination with resveratrol was highly toxic in monolayer culture, with 76% of apoptotic cells at 20ng/ml and 72% in co-treated cultures (Fig 4k).

For investigations in 3-D microenvironment alginate culture, alginate beads were left untreated or treated with 5μM resveratrol and/or with various concentrations of TNF-β, as described in Material and Methods (Fig 5). Untreated or resveratrol treated cultures exhibited viable cells with well-developed cell organelles, organized in well-developed cartilage nodules and embedded in an extensive, fine fibrillar matrix tightly attached to the cytoplasmic membrane. (Fig 5A and 5F). Treatment with TNF-β, lead to dose-dependent increase in morphological signs of degeneration and degradation of chondrocytes such as multiple vacuoles, swelling, degeneration of cell organelles, nuclear fragmentation, formation of apoptotic bodies and detachment between chondrocytes and extracellular matrix (Fig 5B–5E). In contrast, co-treatment with resveratrol protected the chondrocytes from TNF-β-induced cellular degeneration and apoptosis at concentrations from 1 to 10ng/ml, but not at higher concentration of 20ng/ml (Fig 5G–5J). Statistical evaluation of the ultrastructural data of chondrocytes in alginate microenvironment culture displaying severe apoptosis was performed by counting 100 cells from 25 microscopic fields. As shown in Fig 5K TNF-β, effectively induced cell death in 3D microenvironment alginate culture in a dose-dependent manner with 51% apoptotic cells at 10ng/ml, 38% at 5ng/ml, 27% at 1ng/ml compared to 18% in untreated or 16% in resveratrol treated chondrocytes. Apoptosis in resveratrol and TNF-β co-treated cultures was significantly lower with 35% at 10ng/ml, 29% at 5ng/ml and 19% at 1ng/ml TNF-β. Dose of 20ng/ml TNF-β alone or in combination with resveratrol was evidently toxic, with 88% of apoptotic cells at 20 ng/ml and 83% in co-treated cultures (Fig 5K). Taken together, chondrocytes treated with 20ng/ml TNF-β, as opposed to controls or with resveratrol treated cells, were not able to survive in alginate cultures and they underwent cell death.

### Resveratrol suppresses TNF-β-induced inhibition of chondrocyte viability and proliferation in alginate culture

**Fig 6:**
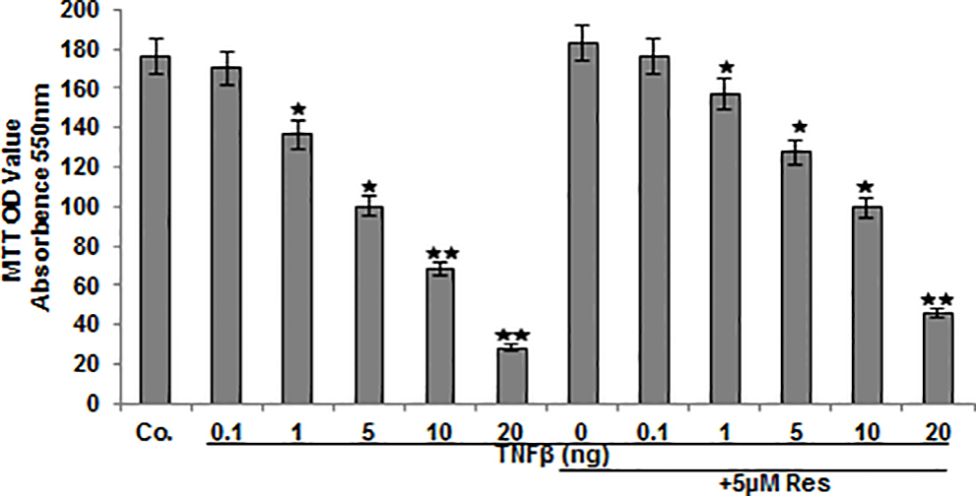
Effects of resveratrol and/or TNF-β on the proliferation of chondrocytes in 3-D alginate culture. Serum-starved human articular chondrocytes were either left untreated or treated with different concentrations of TNF-β alone (0.1, 1, 5, 10, 20ng/ml) or were treated with resveratrol (5 μM) followed by treatment with different concentrations of TNF-β (0, 0.1, 1, 5, 10, 20ng/ml) for 14 days. Cell viability was examined by MTT assay. The MTT assay is a spectrophotometric measurement of the cell viability as a function of the mitochondrial activity. The results are provided as mean values with standard deviations from at least three independent experiments. Values were compared to the control and statistically-significant values with p<0.05 are designated by (*) and p<0.01 designated by (**).

To evaluate cell viability in response to resveratrol and TNF-β treatment, chondrocytes in alginate bead culture were retrieved from alginate and a MTT assay was performed. Alginate beads were left untreated or treated with 5μM resveratrol and/or with various concentrations of TNF-β, as described in detail in Material and Methods. As shown in Fig 6 treatment with lowest concentration of 0.1ng/ml TNF-β had little effect on cell viability compared to untreated cells. In contrast, a significant dose-dependent decrease of 22%, 44%, 60% and 84% in cell viability of chondrocytes treated with 1, 5, 10, and 20ng/ml TNF-β respectively was observed. Cell viability in cells treated with resveratrol alone or combination of resveratrol and 0.1ng/ml TNF-β was similar to untreated cells. Interestingly, it was noted that co-treatment with resveratrol markedly suppressed decrease of 11%, 27%, 44% and 77% in cell viability of chondrocytes treated with 1, 5, 10 and 20 ng/ml TNF-β respectively compared to TNF-β mono-treatment.

### Resveratrol suppresses TNF-β- or T-lymphocytes-induced down-regulation of extracellular matrix, Ki67, Sirt1 and Sox9 in chondrocytes

**Fig 7:**
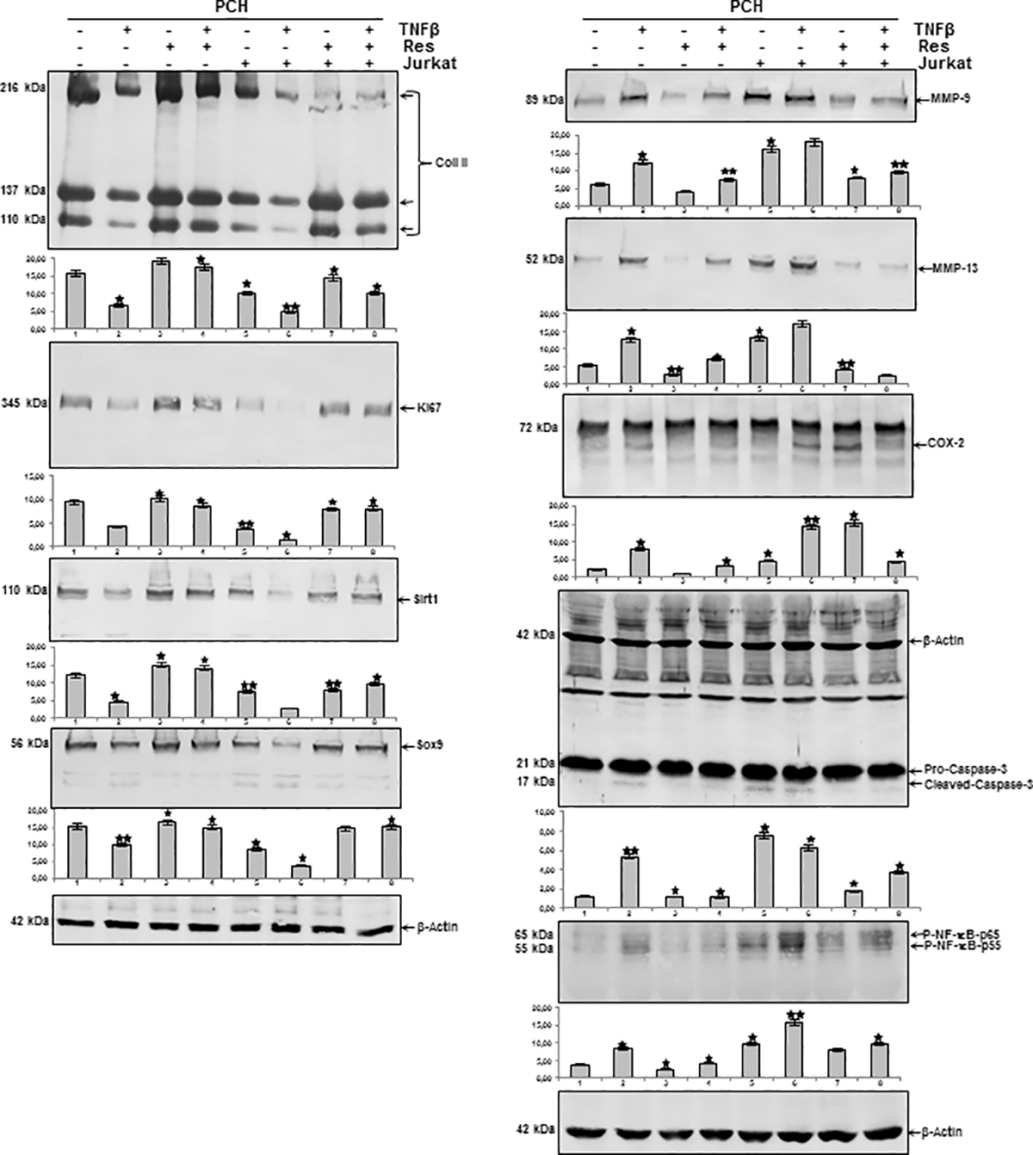
Resveratrol blocks TNF-β- or T-lymphocytes-induced down-regulation of extracellular matrix, Ki67, Sirt1, Sox9, expression of NF-κB and NF-κB-dependent pro-inflammatory, matrix-degrading and apoptotic gene products in chondrocytes. Serum starved chondrocytes were cultured in monolayer and either left untreated or treated with resveratrol, or TNF-β, or co-treated with resveratrol and TNF-β, or co-cultured with T-lymphocytes alone or additionally with TNF-β with or without resveratrol. Whole-cell extracts were prepared and then analyzed by western blotting with indicated antibodies. Densitometric evaluation of protein expression as revealed by western blot analysis was performed in triplicate. *Bars* represent the mean values for collagen type II, Ki67, Sirt1, Sox9, MMP-9, MMP-13, COX-2, cleaved caspase-3 and p-NF-κB. Housekeeping protein β-actin served as a loading control in all experiments. The results are provided as mean values with standard deviations from at least three independent experiments. Values were compared to the control and statistically-significant values with p<0.05 are designated by (*) and p<0.01 designated by (**).

Serum starved chondrocytes were cultured in monolayer and either left untreated or treated with resveratrol, or TNF-β, or co-treated with resveratrol and TNF-β, or co-cultured with T-lymphocytes alone or additionally with TNF-β with or without resveratrol as described in Material and Methods. Western blot analysis was performed by probing whole cell lysates with antibodies against collagen type II, Ki67, Sirt1, the cartilage-specific transcription factor Sox9 and β-actin (Fig 7). As shown in Fig 7, chondrocytes stimulated with TNF-β alone or co-cultured with T-lymphocytes and/or TNF-β showed a significant down-regulation of synthesis of collagen type II, Ki67, Sirt1 and Sox9 expression (Fig 7). In contrast, treatment of chondrocytes with resveratrol followed by stimulation with TNF-β, or co-cultured with T-lymphocytes and/or TNF-β with resveratrol, resulted in a significant inhibition of cytokine-induced effects on the above mentioned protein expression (Fig 7). Interestingly, treatment of the chondrocytes with TNF-β alone or co-culture with T-lymphocytes down-regulated the expression of the above-mentioned proteins in the same manner in chondrocytes and co-culture of the chondrocytes with T-lymphocytes and treatment with TNF-β decreased the levels of the above mentioned-proteins more than each agent by itself (Fig 7). Taken together, these results demonstrate that resveratrol inhibited TNF-β-induced down-regulation of extracellular matrix (ECM) production, chondrocytes proliferation and cartilage-specific transcription factor also in an inflammatory microenvironment with T-lymphocytes, underlining the prophylactic potential of resveratrol to protect the chondrogenic potential in chondrocytes.

### Resveratrol suppresses TNF-β- or T-lymphocytes-induced NF-κB-dependent Pro-inflammatory, matrix-degrading and apoptotic gene products in chondrocytes

We examined further, whether resveratrol can modulate the expression of TNF-β- and/or T-lymphocytes-induced NF-κB-regulated gene products involved in the inflammatory and degradative processes in chondrocytes. Serum starved chondrocytes were left untreated or treated with resveratrol alone and/or with TNF-β, and/or co-cultured with T-lymphocytes as described in Materials and Methods. Equal amounts of total proteins were separated by SDS-PAGE and analyzed by immunoblotting using antibodies raised against MMP-9/-13, COX-2, activated caspase-3 and phosphospecific-p65 as well as β-actin. Fig 7 demonstrates a significant up-regulation of synthesis of MMP-9/-13, cleavage of caspase-3 and phosphorylation of NF-κB in chondrocytes treated with TNF-β and/or co-cultured with T-lymphocytes, compared with basal control (Fig 7). To date, TNF-β induced in a similar way to T-lymphocytes the expression of the above-mentioned proteins in the same manner in chondrocytes and co-culture of the chondrocytes with T-lymphocytes and treatment with TNF-β significantly increased the levels of the above mentioned-proteins more than each agent by itself. Treatment with resveratrol suppressed the expression of the mentioned proteins in all combinations (Fig 7). Taken together, these results further strengthen the role of resveratrol-dependent signaling in modulating TNF-β-induced NF-κB-regulated inflammation in chondrocytes in T-lymphocytes microenvironment.

### Resveratrol suppresses down-regulation of extracellular matrix, Ki67, Sirt1 and Sox9 in chondrocytes induced by TNF-β or NAM but not by ASO against Sirt1

**Fig 8:**
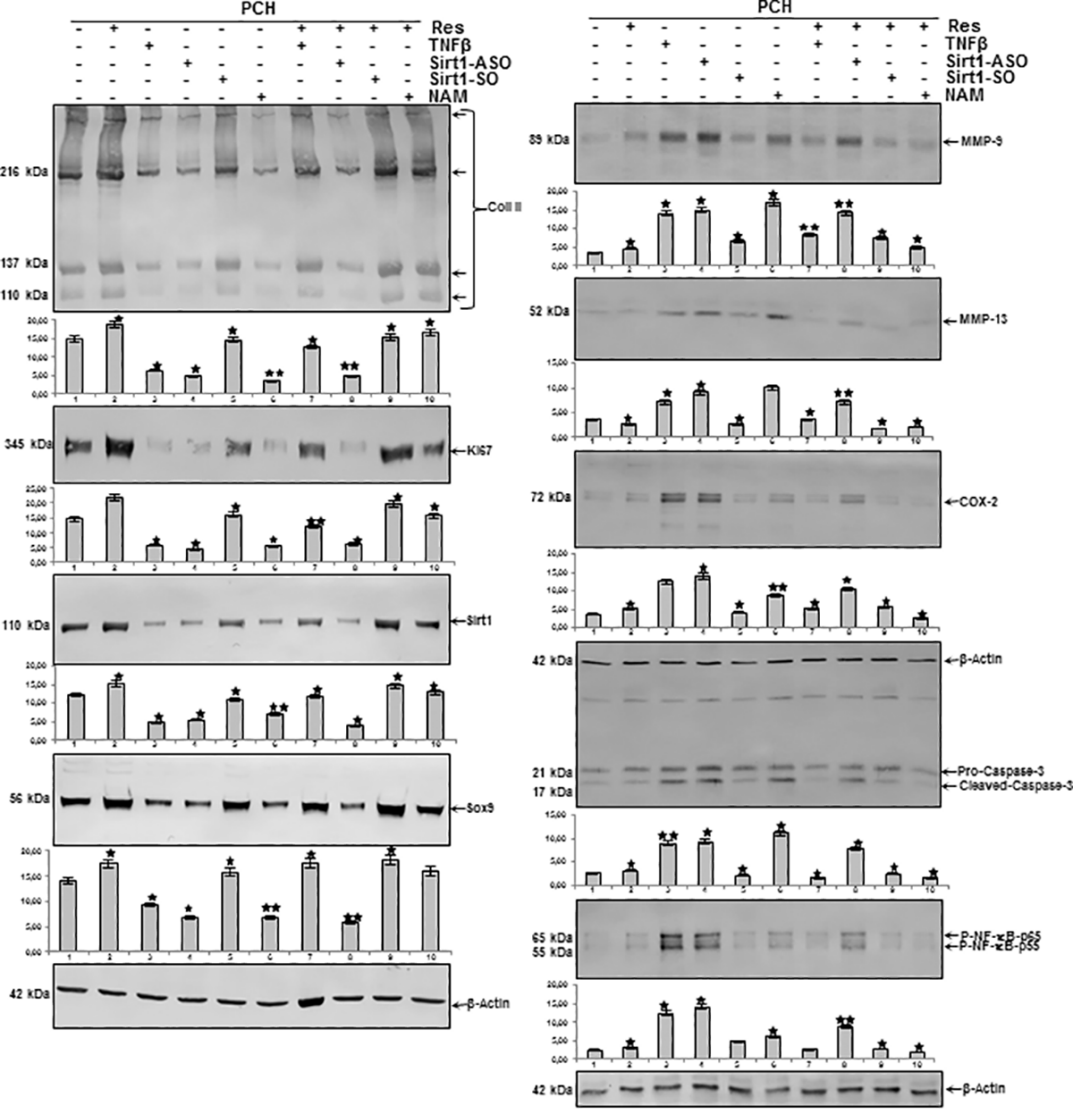
Effects of resveratrol, TNF-β, NAM, ASO against Sirt-1 on extracellular matrix, Ki67, Sirt1, Sox9, NF-κB, NF-κB-dependent pro-inflammatory, matrix-degrading and apoptotic gene products in chondrocytes. Serum starved chondrocytes in monolayer culture were either left untreated or treated with resveratrol (5μM), TNF-β (10ng/ml), or with Sirt1-SO or -ASO (0.5μM) in the presence of Lipofectin for 24h, or NAM (10mM), or cells were pretreated with resveratrol (5μM) for 1h followed by co-treatment with TNF-β (10ng/ml), Sirt1-SO, or -ASO (0.5μM) in the presence of Lipofectin for 24h, NAM (10mM) as described in Material and Methods. Whole cell extracts were prepared, separated by SDS-PAGE, and subjected to western blot analysis using relevant antibodies. Densitometric evaluation of protein expression as revealed by western blot analysis was performed in triplicate. *Bars* represent the mean values for collagen type II, Ki67, Sirt1, Sox9, MMP-9, MMP-13, COX-2, cleaved caspase-3 and p-NF-κB. Housekeeping protein β-actin served as a loading control in all experiments. The results are provided as mean values with standard deviations from at least three independent experiments. Values were compared to the control and statistically-significant values with p<0.05 are designated by (*) and p<0.01 designated by (**).

As shown above, resveratrol/Sirt1-dependently blocked TNF-β- and/or T-lymphocytes-induced inflammation and degenerative changes in PCH. To investigate further the role of Sirt1 in TNF-β-induced degradative and degenerative changes in chondrocytes in an inflammatory microenvironment, chondrocytes were either left untreated or treated with resveratrol, TNF-β, NAM and/or transfected with Sirt1-ASO or–SO as described in Materials and Methods. Equal amounts of total proteins were separated by SDS-PAGE and analyzed by immunoblotting using antibodies raised against collagen type II, Ki67, Sirt1, Sox9 and β-actin. As shown in Fig 8, there was a significant down-regulation of synthesis of collagen type II, Ki67, Sirt1 and Sox9 expression in chondrocytes treated with TNF-β or NAM and ASO against Sirt1, compared with basal control, resveratrol and Sirt1-SO treatment. In contrast, co-treatment with resveratrol up-regulated the above mentioned protein expression to control levels in all treatment groups, except in cells treated with Sirt1-ASO (Fig 8). These results demonstrate an essential role for Sirt1 in TNF-β-mediated inflammatory effects in cartilage and underline the prophylactic potential of resveratrol as natural Sirt1-activator to protect the chondrogenic potential in chondrocytes.

### Resveratrol suppresses up-regulation of NF-κB-dependent pro-inflammatory, matrix-degrading and apoptotic gene products in chondrocytes induced by TNF-β- or NAM but not by ASO against Sirt1

Next, we examined further the role of Sirt1 on TNF-β-induced inflammatory pathways in PCH. Equal amounts of total proteins were separated by SDS-PAGE and analyzed by immunoblotting using antibodies raised against MMP-9/-13, COX-2 and activated caspase-3 as well as β-actin. As shown in Fig 8, a significant up-regulation of synthesis of MMP-9/-13, cleavage of caspase-3 and phosphorylation of NF-κB in chondrocytes treated with TNF-β or NAM and ASO against Sirt1, compared with basal control, resveratrol and Sirt1-SO treatment. In contrast, co-treatment with resveratrol down-regulated the above mentioned protein expression to control levels in all treatment groups, except in cells treated with Sirt1-ASO (Fig 8). Taken together, these findings suggest that inhibition of Sirt1 on the protein level by Sirt1 inhibitor NAM and on the mRNA level by ASO equally produces an inflammatory response as by stimulation with pro-inflammatory cytokine TNF-β and that resveratrol can suppress this inflammation signaling through activation of Sirt1, if inhibition of Sirt1 is induced at the protein level and not at the gene level.

### Resveratrol modulates TNF-β-induced inflammation and apoptosis in human chondrocytes in 3-D alginate cultures *in vitro*

Several studies suggest that in alginate culture only cells with chondrogenic potential can survive and alginate functions as a selective filter station separating vital from nonvital chondrocytes. The alginate environment appears to be an ideal model for the study of chondrocyte differentiation and matrix components in a three-dimensional system [45, 52].

To examine, whether resveratrol blocks the TNF-β-induced inhibition of extracellular matrix, Ki67, Sirt1, Sox9 and up-regulation of NF-κB-dependent inflammatory pathways and apoptosis, serum-starved chondrocytes in alginate beads were left untreated or treated with various concentrations of TNF-β or were co-stimulated with resveratrol and various concentrations of TNF-β for 14 days as described in Material and Methods.

#### A: Resveratrol suppresses down-regulation of extracellular matrix, Ki67, Sirt1 and Sox9 in chondrocytes induced by TNF-β in 3-D alginate culture

**Fig 9:**
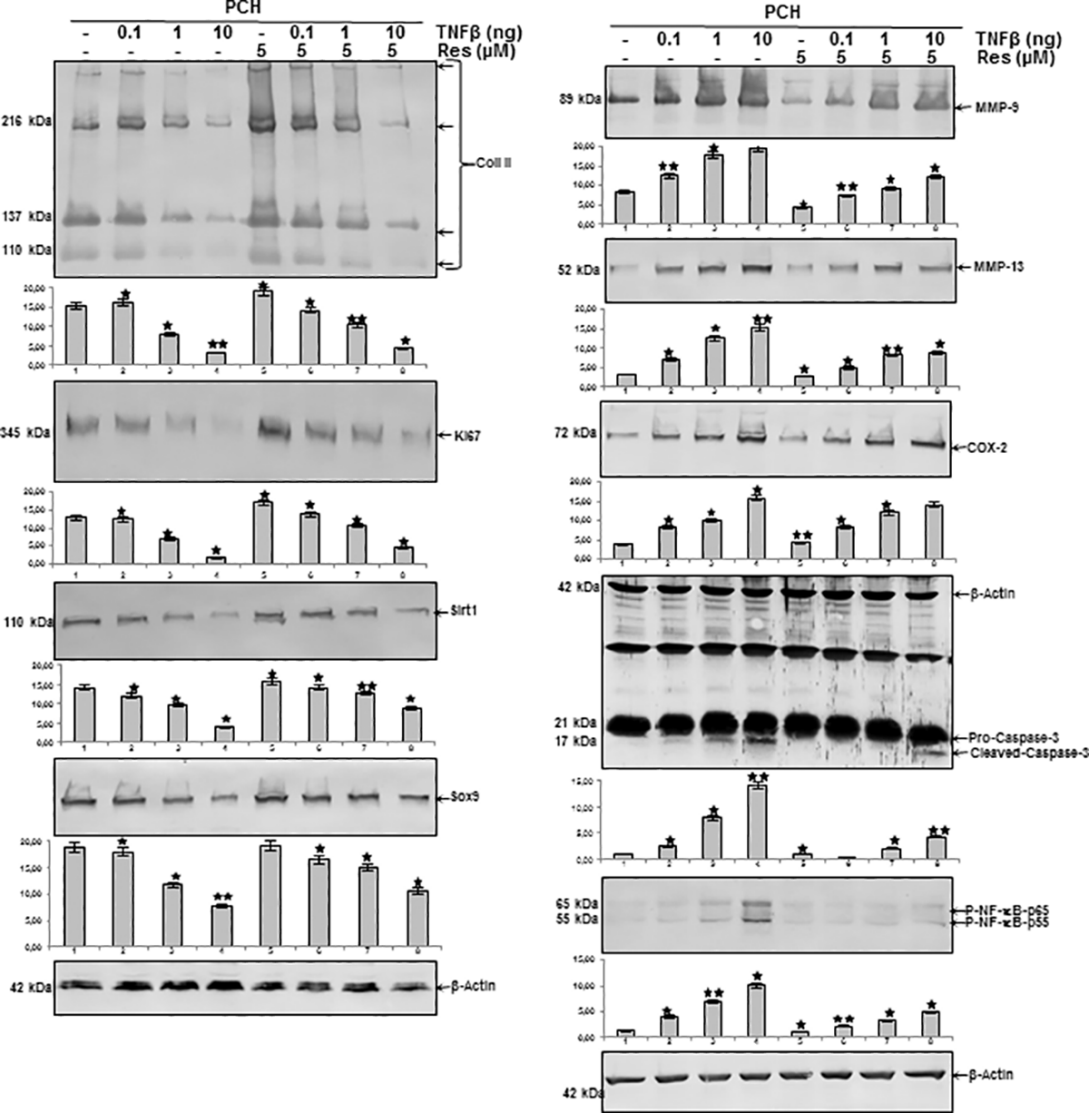
Effects of resveratrol on TNF-β-induced inflammation and apoptosis in chondrocytes in 3D-alginate cultures. Serum-starved chondrocytes in alginate beads were left untreated or treated with various concentrations of TNF-β or were co-stimulated with resveratrol and various concentrations of TNF-β for 14 days as described in Material and Methods. Whole cell lysates were fractionated and subjected to western blotting with indicated antibodies. Densitometric evaluation of protein expression as revealed by western blot analysis was performed in triplicate. *Bars* represent the mean values for collagen type II, Ki67, Sirt1, Sox9, MMP-9, MMP-13, COX-2, cleaved caspase-3 and p-NF-κB. Housekeeping protein β-actin served as a loading control in all experiments. The results are provided as mean values with standard deviations from at least three independent experiments. Values were compared to the control and statistically-significant values with p<0.05 are designated by (*) and p<0.01 designated by (**).

Chondrocytes in 3-D alginate microenvironment stimulated with TNF-β alone showed a significant down-regulation of synthesis of collagen type II, Ki67, Sirt1 and Sox9 in a dose-dependent manner, compared with basal control and resveratrol (Fig 9). At a concentration of 10ng/ml the mentioned proteins were almost completely inhibited. In contrast, co-treatment with resveratrol up-regulated the above mentioned protein expressions to control levels in all TNF-β-treated groups, expect in cells treated with 10ng/ml TNF-β and this was concentration dependent (Fig 9).

#### B: Resveratrol suppresses up-regulation of NF-κB-dependent pro-inflammatory, matrix-degrading and apoptotic gene products in chondrocytes induced by TNF-β in 3-D alginate culture

Whole cell lysates were fractionated and analyzed by immunoblotting using anti-MMP-9/-13, anti-COX-2 and anti-activated caspase-3 as well as β-actin. As shown in Fig 9, in western blot analysis, treatment with TNF-β clearly up-regulated the expression of the mentioned proteins in a dose-dependent manner. In contrast, co-treatment with resveratrol significantly decreased the expression of the mentioned proteins in a concentration-dependent manner to levels similar to control cultures (Fig 9). Taken together, these results further confirmed the role of resveratrol-dependent signaling in modulating TNF-β-induced NF-κB-regulated gene products, and further suggest that TNF-β is one of the major cytokines mediating inflammations in RA/OA microenvironment.

## Discussion

The goal of this study was to examine the inflammatory role of lymphotoxin α (TNF-β) in an inflammatory microenvironment of primary human chondrocytes and T-lymphocytes *in vitro* and to determine the anti-inflammatory potential of resveratrol signaling to suppress inflammatory pathways activated by TNF-β in chondrocytes in monolayer and 3D alginate cultures.

We have made the following novel findings: (I) TNF-β-mediated adhesiveness of T-lymphocytes to chondrocytes could be significantly reduced by resveratrol and this effect could be inhibited by knock-down of Sirt1 with ASO, (II) resveratrol suppressed NF-κB activation induced by TNF-β or T-lymphocytes in chondrocytes, (III) resveratrol also down-regulated NF-κB dependent gene products involved in cell proliferation (e.g. Ki67), inflammatory (COX-2), in matrix degrading (MMP-9/-13) and apoptotic (cleaved caspase-3) signaling pathways induced by TNF-β- or T-lymphocytes in the same manner, (IV) this downregulation led to the suppression of apoptosis induced by cytokines and T-lymphocytes, (V) Finally, we showed for the first time, that TNF-β- similar to T-lymphocytes-mediated inflammation was inhibited by resveratrol in a Sirt1-dependent manner, highlighting the crucial role of this enzyme in regulation of TNF-β-induced inflammation signaling in chondrocytes.

Rheumatoid arthritis (RA) is an inflammatory joint disease affecting 0.5–1% of adults, with 5–50 per 100 000 new cases annually [2]. Indeed, recruitment of lymphocytes to the site of inflammation plays a pivotal role in mediating inflammation in RA [47]. It is well characterized that overproduction and overexpression of TNF-α acts as a key initiator in the inflammatory cascade, activating members of the Interleukin-family such as Interlekuin-1β (IL-1β), enhancing interactions between T-and B-lymphocytes, synovial-like fibroblasts and macrophages, leading to synovial inflammation and joint destruction [3, 53]. However, not all adverse effects in RA can be related to TNF-α signaling, as approximately in 30% of patients treatment with biologic therapy (typically initiated with TNF-α blockers) fails, placing them at risk of further cartilage and joint damage [54]. Importantly, this indicates the participation of other cytokines in the early pathogenesis of RA. Interestingly, an increasing body of evidence suggested that the TNF-α homologue Lymphotoxin α, alias TNF-β, plays a pivotal role in inflammatory joint environment [8, 12, 13]. Indeed, a recent report from our laboratory revealed that in an *in vitro* model of inflammatory joint environment TNF-β enhanced inflammation in chondrocytes, similar to IL-1β [14].

We showed in this study for the first time that TNF-β, similar to TNF-α enhances recruitment and adherence of T-lymphocytes to chondrocytes, underling the potential of TNF-β in stimulating and supporting an inflammatory microenvironment in chondrocytes. Indeed, it has been previously shown that an allelic variant in the TNF-β gene leads to higher TNF-β response in mononuclear cells after stimulation with mitogens [55]. The authors assumed that these allelically varying TNF-β responses of activated T-lymphocytes might contribute to the slow and self-protruding inflammatory mechanisms of local autoimmune reactions and this might be relevant for MHC-associated predispositions for autoimmune diseases [55]. Interestingly, recently Shaker *et al*. have further shown that polymorphisms in the TNF-β gene increase susceptibility to RA [56].

Additionally, in this study, we evaluated for the first time that co-treatment with the natural polyphenol resveratrol suppressed T-lymphocytes adherence to chondrocytes in TNF-β as well as in T-lymphocytes treated cultures and this effect of resveratrol was inhibited by knock-down of Sirt1 with antisense oligonucleotides (Sirt1-ASO). This demonstrates that inflammation in chondrocytes by TNF-β is indeed mediated similar to inflammation by T-lymphocytes. Further, this indicates clearly, that resveratrol-induced suppression of inflammatory microenvironment by TNF-β or T-lymphocytes in chondrocytes is, at least in part, dependent on activation of Sirt1 pathway. These results are consistent with those from previous studies that showed that the multi-targeted, natural polyphenol compound resveratrol has been linked with anti-inflammatory and chondroprotective properties in chondrocytes [21–23] and further has been shown to significantly up-regulate the NAD+-dependent Histondeacetylase Sirt1 in normal and osteoarthritic chondrocytes [24, 25]. Furthermore, it has been well studied, that activation of Sirt1 plays a pivotal role in chondrocytes differentiation and encourages survival under stress conditions [25, 29]. Moreover, this could explain why TNF-α induced inflammation leads to Sirt1 overexpression in synovial tissue of RA patients [27].

To determine the mechanisms by which the pro-inflammatory signaling of TNF-β in enhancing adhesiveness of T-lymphocytes to chondrocytes and the activation of inflammation cascade in chondrocytes is regulated, we next investigated with immunofluorescence, how TNF-β-modulated activation of NF-κB and Sirt1 in comparison to T-lymphocytes effects. Indeed, it has been shown in chondrocytes that activated NF-κB is involved in regulation of adhesion, cell-cycle, apoptosis, survival and inflammatory processes by activating IL-1β, TNF-α, IL-6, Cox-2 and MMPs [10, 11, 57, 58]. For activation of NF-κB dependent pro-inflammatory signaling cascade, following phosphorylation and ubiquitination, the p65 and p50 subunits dissociate from the IκBα complex and translocate to the nucleus to bind to NF-κB recognition sites in the promoter regions [50]. Further, it has been already reported that TNF-β possesses similar but less potent inflammatory potential than TNF-α [59]. Interestingly, in a recent report, we could show that TNF-β induced activation of NF-κB similar to IL-1β in primary human chondrocytes [14]. In this report, we found now for the first time that indeed, TNF-β, similar to T-lymphocytes, induced activation and nuclear translocation of NF-κB and concomitantly suppressed Sirt1 activation. Furthermore, we could show that resveratrol co-treatment with either TNF-β or T-lymphocytes significantly up-regulated Sirt1 and concomitantly blocked NF-κB activation and nuclear translocation in chondrocytes. In fact, several lines of evidence have shown that resveratrol is a suppressor of the NF-κB transcription factor [60, 61] and a potent activator of Sirt1 in chondrocytes [24, 48]. These results highlight the crucial role of Sirt1 in inhibiting T-lymphocytes- or TNF-β-induced pro-inflammatory microenvironment by the NF-κB pathway in chondrocytes.

We have previously shown that untreated primary chondrocytes have very low TNF-β expression and that stimulation with a pro-inflammatory cytokine like IL-1β induced TNF-β in chondrocytes [14]. However, the molecular mechanism(s) of TNF-β on chondrogenic potential, cell viability, chondrocytes morphology, cell-cell contacts or cell-matrix interactions of chondrocytes has not been explored. Here, we now demonstrate with ultrastructural investigations and MTT-assay that indeed, up-regulation of inflammatory signaling pathways by TNF-β are associated with reduced cell viability, degenerative cell morphology changes, loss of cell-cell and cell-matrix interaction and finally resulting in apoptosis. Interestingly, these morphological alterations are similar to those described for degenerative and inflammatory activation in chondrocytes by TNF-α and IL-1β [22, 58, 62]. We further showed that resveratrol treatment protected the cells from TNF-β-induced degradative and apoptotic morphological alterations up to certain concentrations of TNF-β. These results are in accordance with results from previous studies from our laboratory and others underling the potential of resveratrol to suppress pro-inflammatory signaling in chondrocytes and exhibit a chondroprotective effect [21, 23, 37].

To examine the underlying downstream signaling pathway, by which resveratrol executes its inhibitory effect on TNF-β-signaling pathway, we investigated whether NF-κB and NF-κB-regulated gene end-products involved in inflammation, proliferation and apoptosis were activated. We found that resveratrol significantly suppressed NF-κB activation induced by TNF-β and/or T-lymphocytes in chondrocytes. NF-κB suppression correlated with reduced p65 phosphorylation and nuclear translocation. Furthermore, resveratrol also down-regulated NF-κB dependent gene end-products involved in cell proliferation (e.g. Ki67), inflammatory (COX-2), in matrix degrading (MMP-9/-13) and apoptotic (cleaved caspase-3) signaling pathways induced by TNF-β- or T-lymphocytes. Interestingly, the inhibition effect of resveratrol was, at least in part, Sirt1-signalling dependent. Resveratrol’s inhibition of the NF-κB activation induced by TNF-β and/or T-lymphocytes suggests that resveratrol/Sirt1 signaling acts at a step common to all of these activators. However, we are the first to report the ability of resveratrol to modulate expression and activation of TNF-β in chondrocytes and TNF-β-induced RA-microenvironment. These results suggest that the effects of resveratrol on TNF-β signaling are, at least in part, through suppression of NF-κB signaling in chondrocytes.

We further found that treatment of chondrocytes with ASO-Sirt1, like NAM or TNF-β, caused NF-κB activation and up-regulation of NF-κB-related gene end-products involved in degradation, inflammation and apoptosis (MMP-9/-13, COX-2 expression, caspase-3 cleavage) and this was accompanied by the decreased synthesis of cartilage-specific matrix, Ki67, Sirt1 and Sox9 expression. However, co-treatment with resveratrol inhibited NF-κB activation and NF-κB-related gene end-products by NAM, TNF-β or T-lymphocytes and revealed a significant increasing in cartilage-specific matrix expression, Ki67, Sirt1 and Sox9 expression, but not by Sirt1-ASO. These results show clearly that TNF-β-mediated NF-κB activation in chondrocytes is similar to Sirt1-ASO or NAM, indicating that Sirt1 is one of the main downstream signaling molecules for TNF-β-signaling that may be modulated by resveratrol-mediated signaling pathways in RA inflammatory microenvironment. Moreover, these findings clearly indicate that down-regulation of Sirt1 by mRNA interference abrogates the suppressive effects of resveratrol on TNF-β- and NF-κB signaling, highlighting the important role and function of Sirt1 for anti-inflammatory and anti-cytokine pathway of resveratrol in RA/OA inflammatory joint environment.

## Conclusions

**Fig 10:**
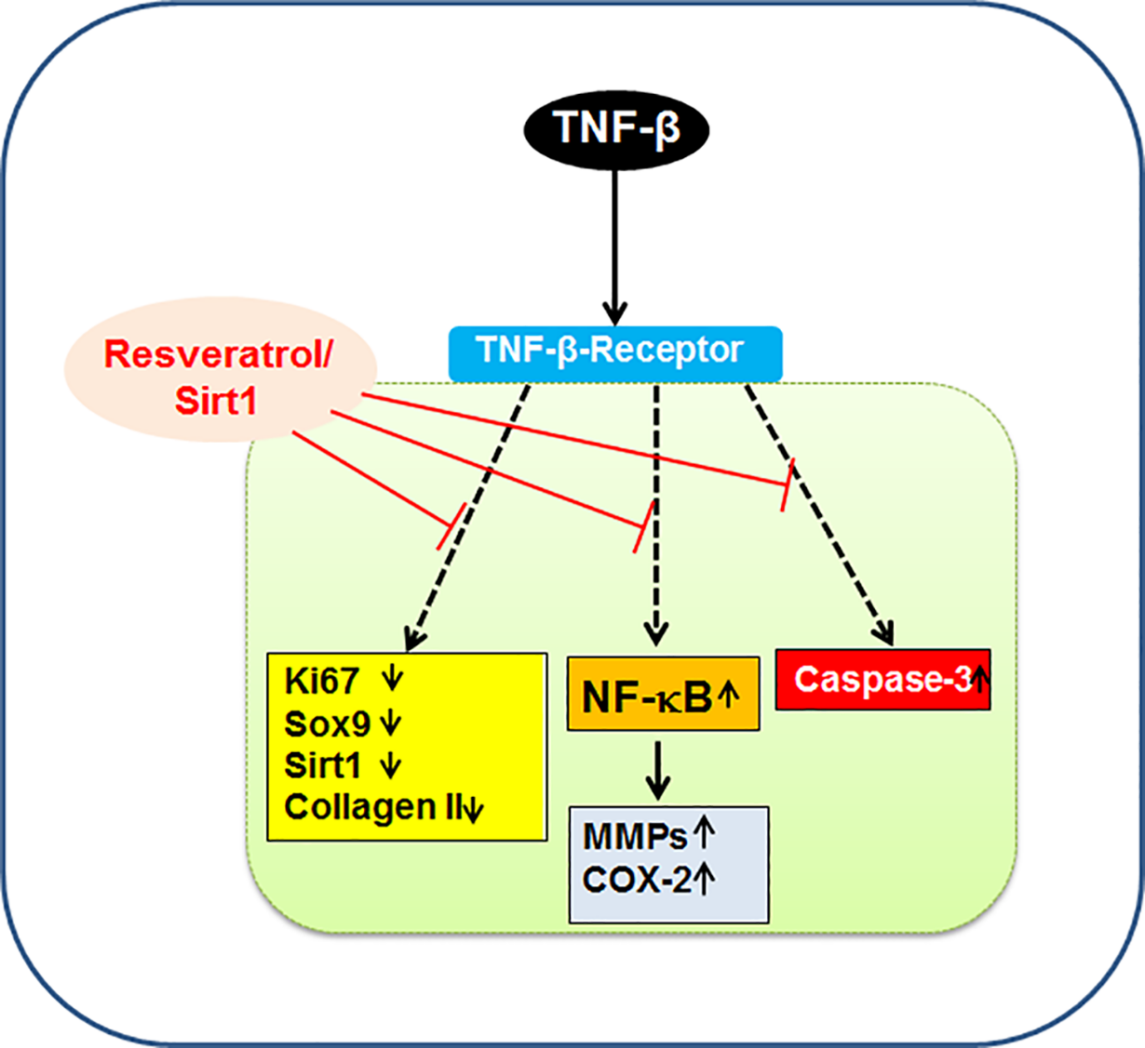
Schematic diagram illustrating TNF-β-induced up-regulation of pro-inflammatory, apoptotic signaling and suppression of cell viability, chondrogenic markers modulated by resveratrol-Sirt1 signaling pathways.

In conclusion, our study here demonstrates for the first time that TNF-β induces a pro-inflammatory microenvironment similar to TNF-α and T-lymphocytes, up-regulating pro-inflammatory signaling and suppressing chondrogenic potential in primary human chondrocytes. Further, we could demonstrate that resveratrol-mediated Sirt1 up-regulation inhibits TNF-β-induced pro-inflammatory and degenerative pathways in chondrocytes (Fig 10). Thus, targeting TNF-β might be a novel and promising therapeutic approach for improving and supporting anti-inflammatory therapy in RA/OA.

## Supporting information

S1 Fig Original western blots to Fig 7. (ZIP)

S2 Fig Original western blots to Fig 8. (ZIP)

S3 Fig Original western blots to Fig 9. (ZIP)
