# Supplementary figures and images for: Resveratrol downregulates inflammatory pathway activated by lymphotoxin α (TNF-β) in articular chondrocytes: Comparison with TNF-α
Source: PLoS One. 2017 Nov 2;12(11):e0186993. doi: 10.1371/journal.pone.0186993 (PMC5667866; doi:10.1371/journal.pone.0186993)

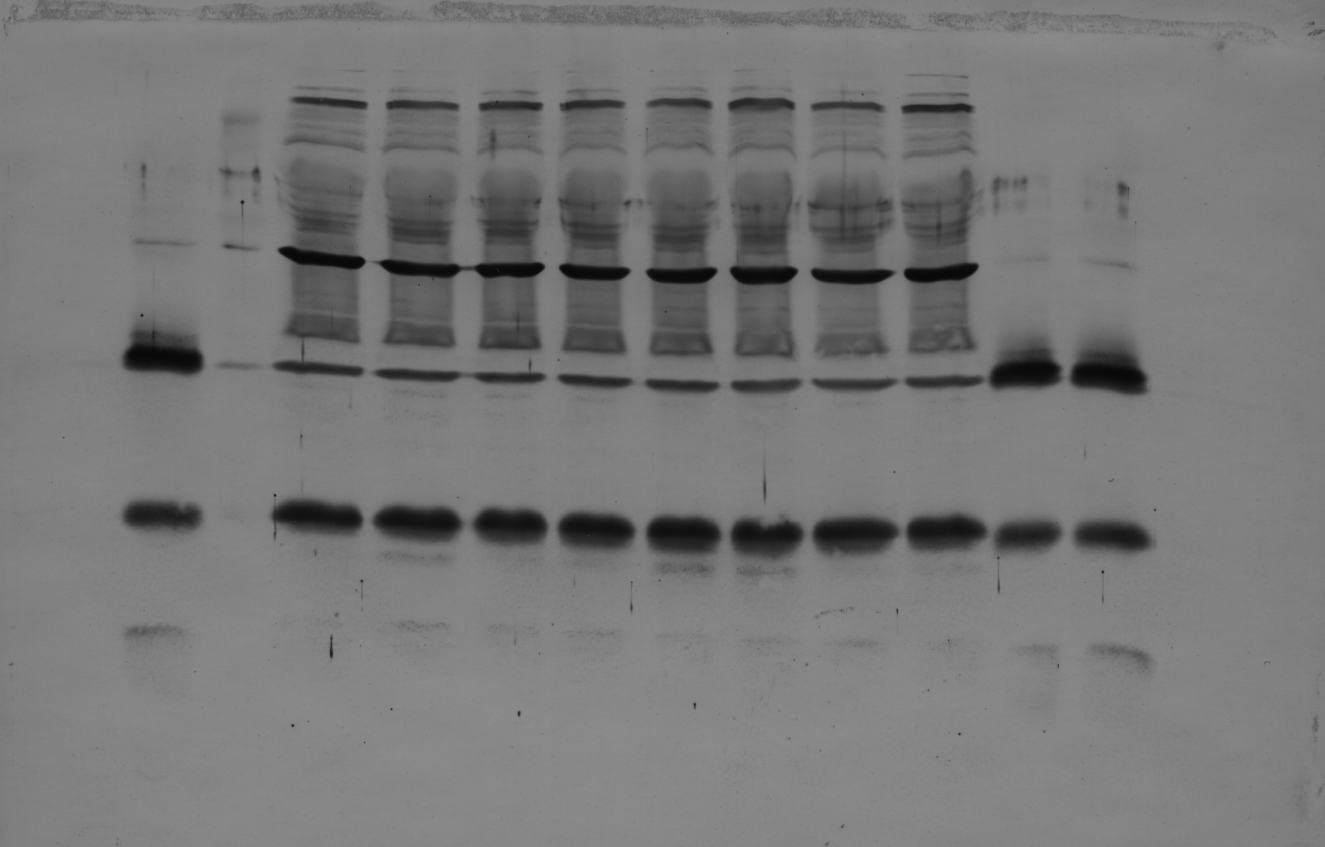

Supplement: S1 Fig — (ZIP) [file pone.0186993.s001.zip › Fig.7-Caspase-3-Actin-PLoSONE-Original.tif]

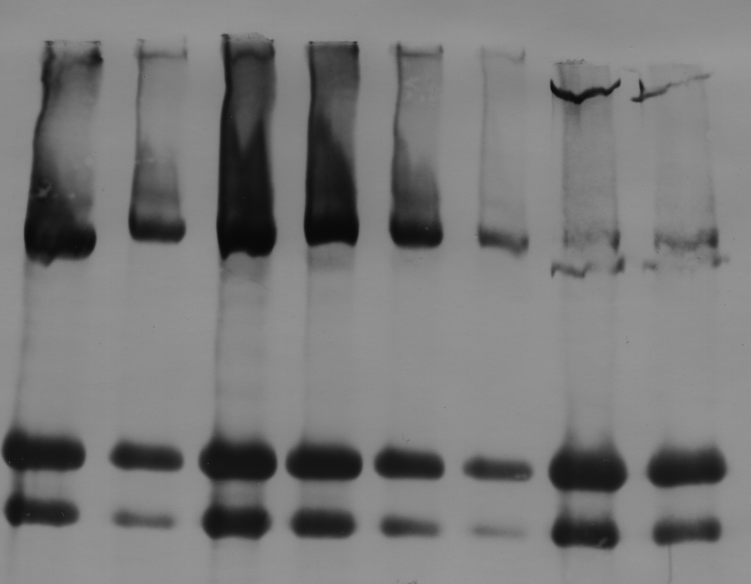

Supplement: S1 Fig — (ZIP) [file pone.0186993.s001.zip › Fig.7-Collagen II-PLoSONE-Original.tif]

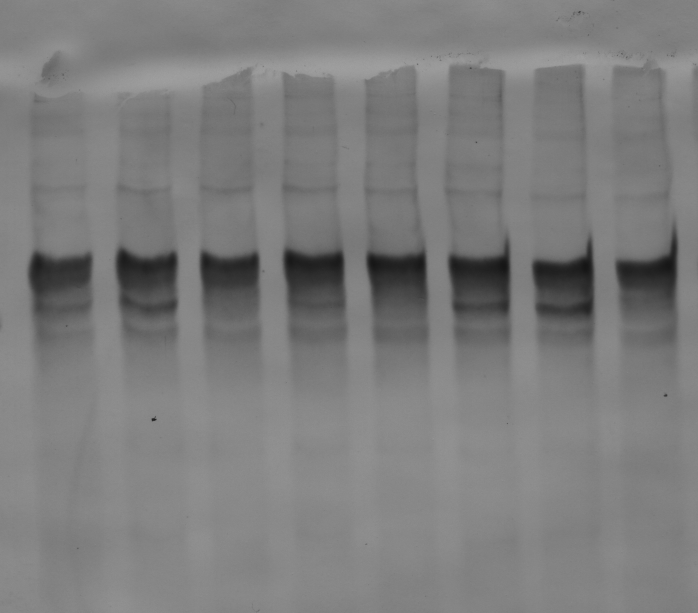

Supplement: S1 Fig — (ZIP) [file pone.0186993.s001.zip › Fig.7-COX2-PLoSONE-Original.tif]

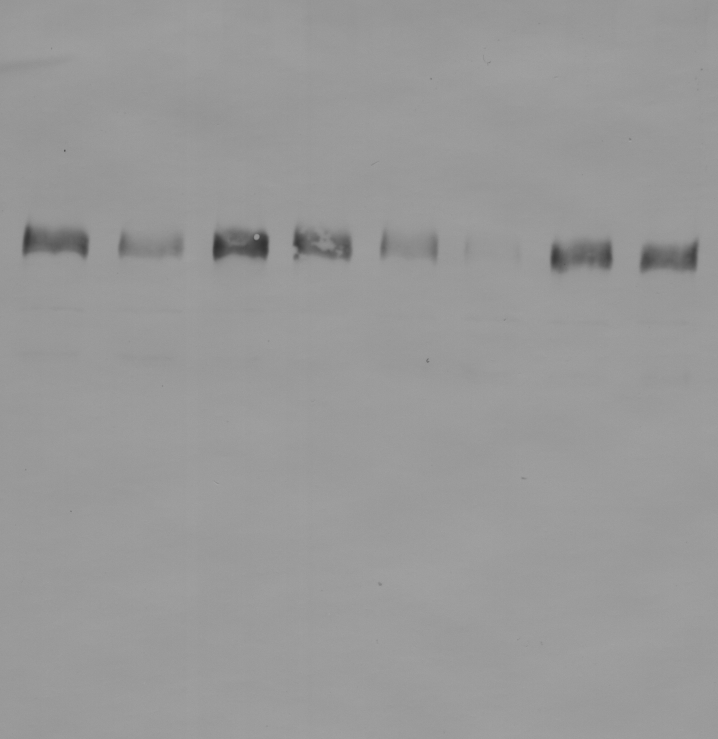

Supplement: S1 Fig — (ZIP) [file pone.0186993.s001.zip › Fig.7-Ki67-PLoSONE-Original.tif]

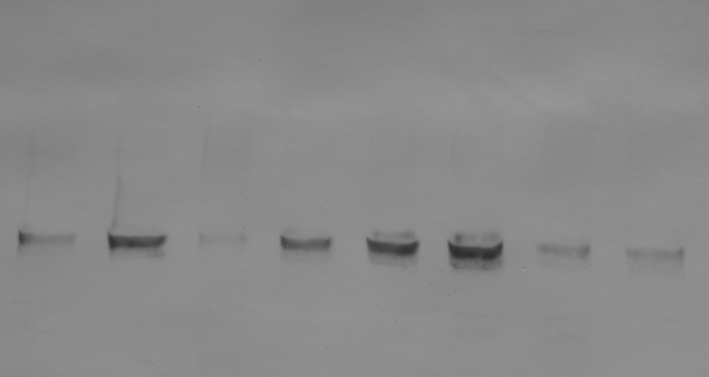

Supplement: S1 Fig — (ZIP) [file pone.0186993.s001.zip › Fig.7-MMP-13-PLoSONE-Original.tif]

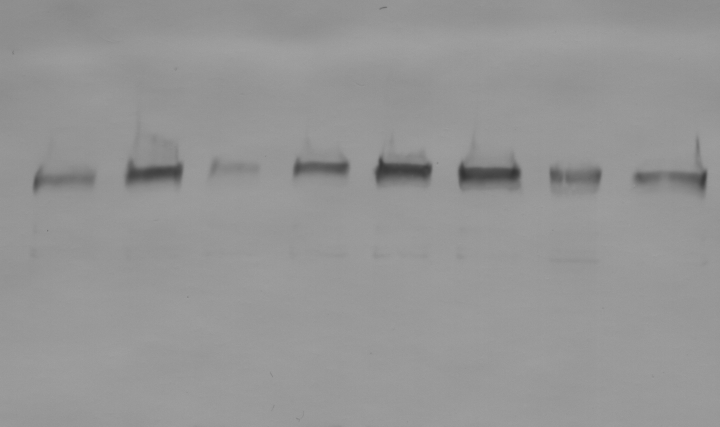

Supplement: S1 Fig — (ZIP) [file pone.0186993.s001.zip › Fig.7-MMP-9-PLoSONE-Original.tif]

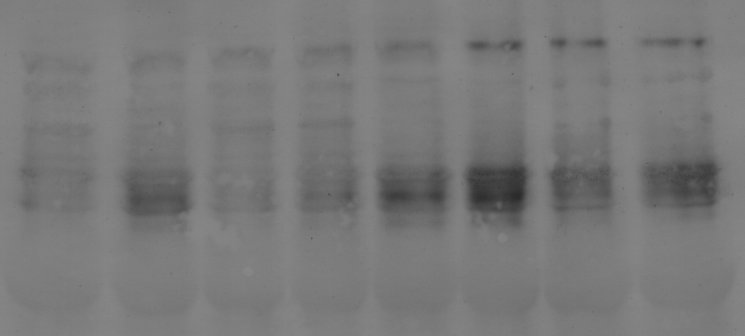

Supplement: S1 Fig — (ZIP) [file pone.0186993.s001.zip › Fig.7-NF-kB-PLoSONE-Original.tif]

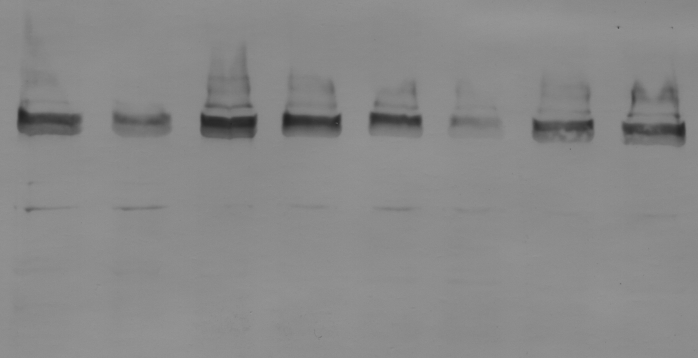

Supplement: S1 Fig — (ZIP) [file pone.0186993.s001.zip › Fig.7-Sirt1-PLoSONE-Original.tif]

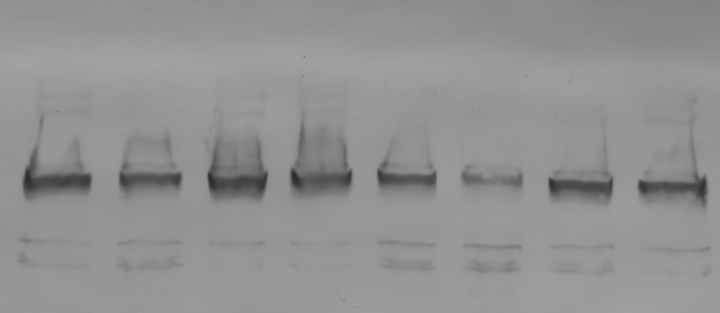

Supplement: S1 Fig — (ZIP) [file pone.0186993.s001.zip › Fig.7-Sox9-PLoSONE-Original.tif]

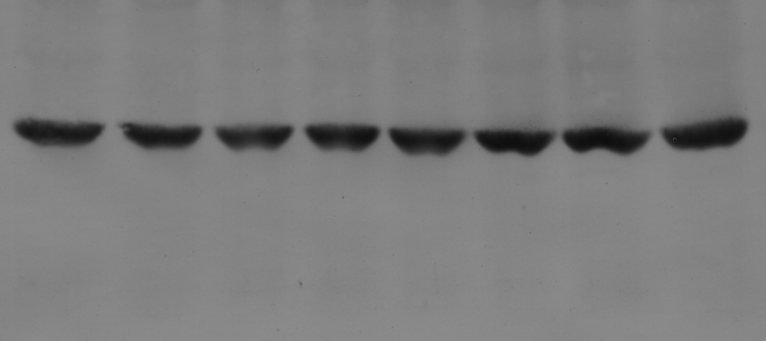

Supplement: S1 Fig — (ZIP) [file pone.0186993.s001.zip › Fig.7-ß-Actin-PLoSONE-Original.tif]

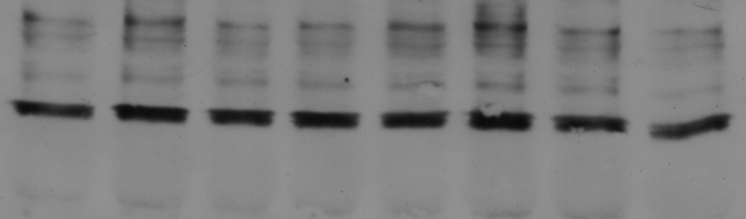

Supplement: S1 Fig — (ZIP) [file pone.0186993.s001.zip › Fig.7-ß-Actin1-PLoSONE-Original.tif]

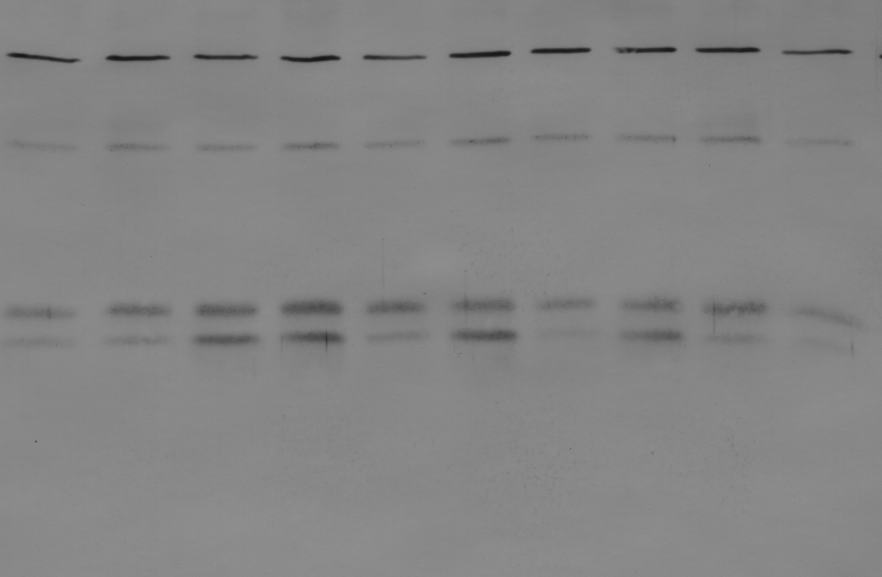

Supplement: S2 Fig — (ZIP) [file pone.0186993.s002.zip › Fig.8-Caspase-3-Actin-PLoSONE-Original.tif]

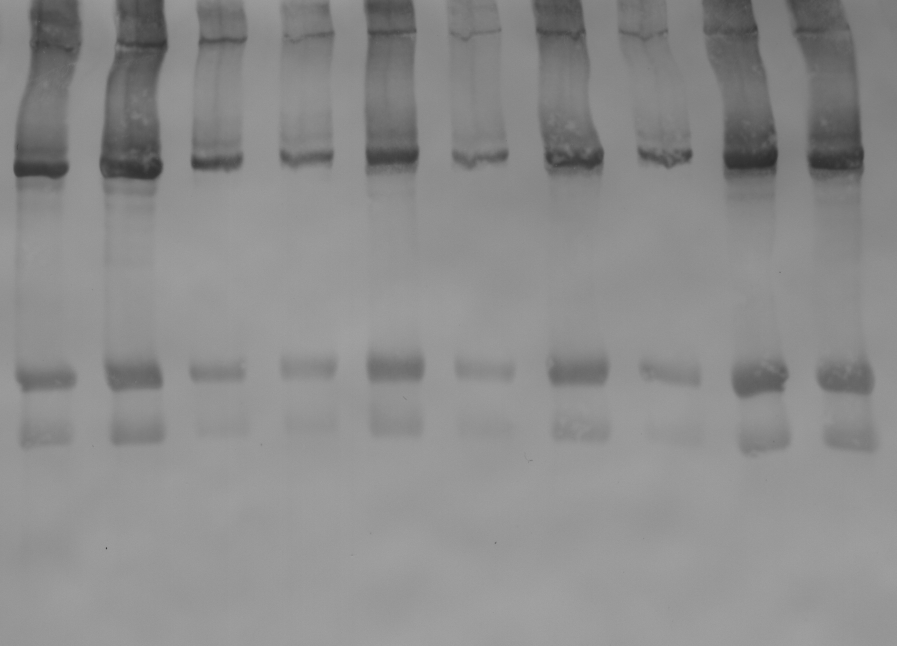

Supplement: S2 Fig — (ZIP) [file pone.0186993.s002.zip › Fig.8-Collagen II-PLoSONE-Original.tif]

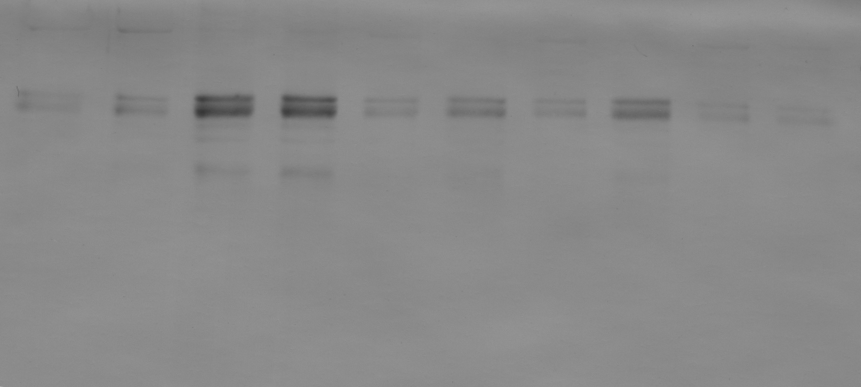

Supplement: S2 Fig — (ZIP) [file pone.0186993.s002.zip › Fig.8-COX-2-PLoSONE-Original.tif]

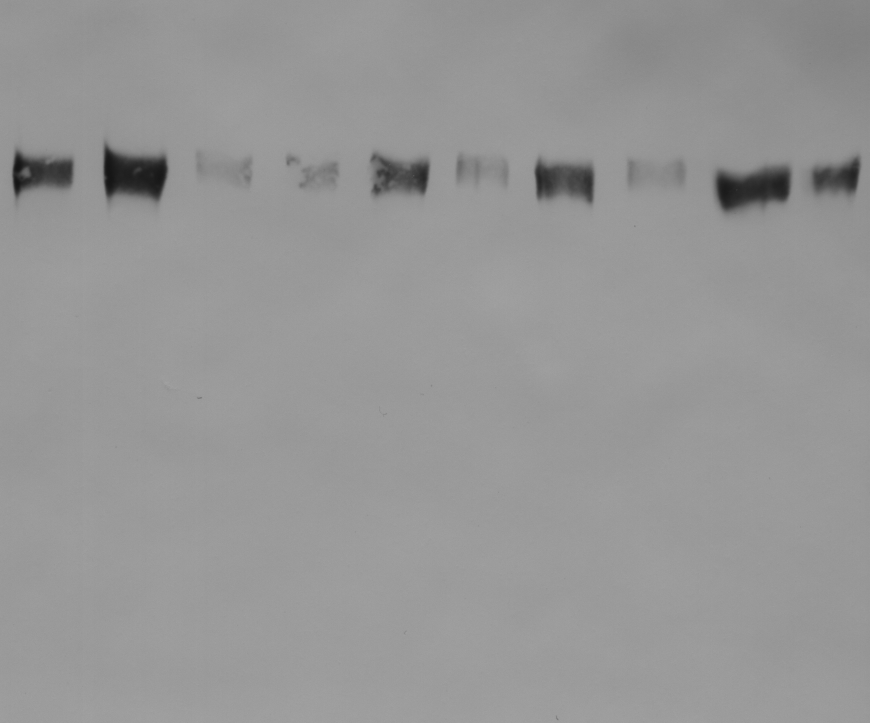

Supplement: S2 Fig — (ZIP) [file pone.0186993.s002.zip › Fig.8-Ki67-PLoSONE-Original.tif]

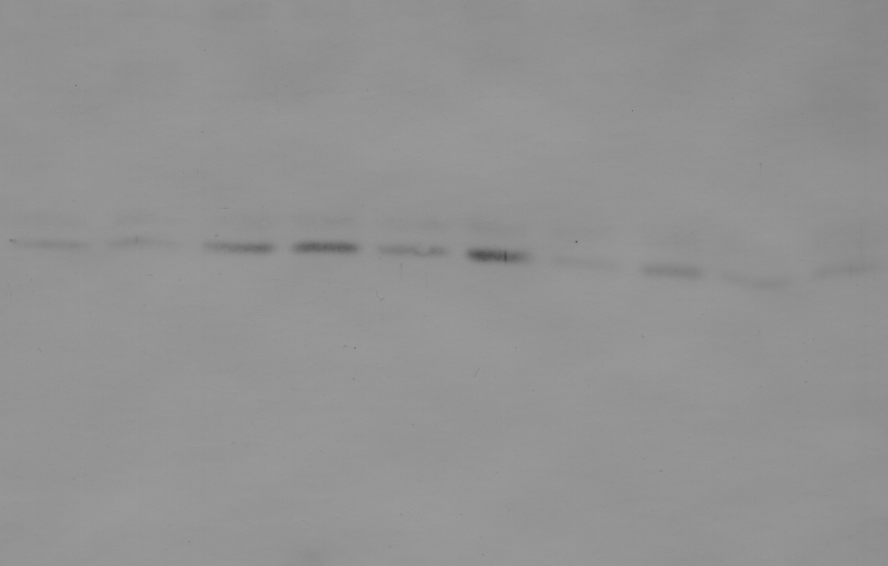

Supplement: S2 Fig — (ZIP) [file pone.0186993.s002.zip › Fig.8-MMP-13-PLoSONE-Original.tif]

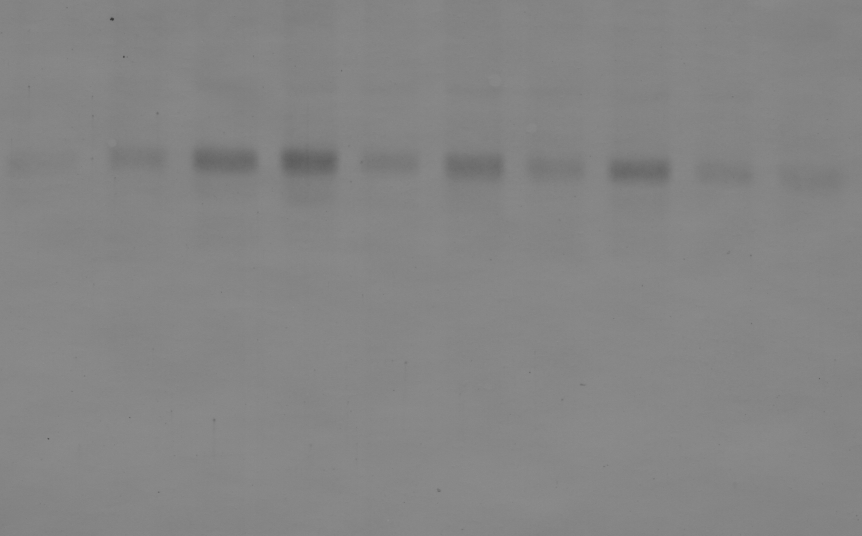

Supplement: S2 Fig — (ZIP) [file pone.0186993.s002.zip › Fig.8-MMP-9-PLoSONE-Original.tif]

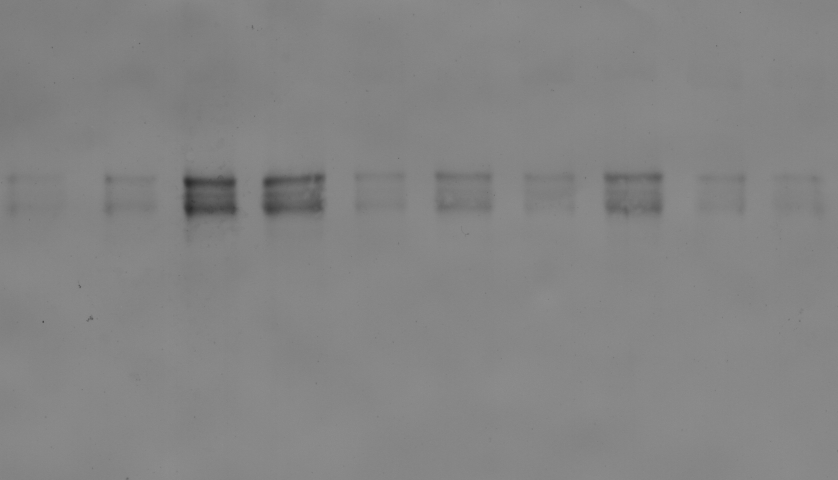

Supplement: S2 Fig — (ZIP) [file pone.0186993.s002.zip › Fig.8-NF-kB-PLoSONE-Original.tif]

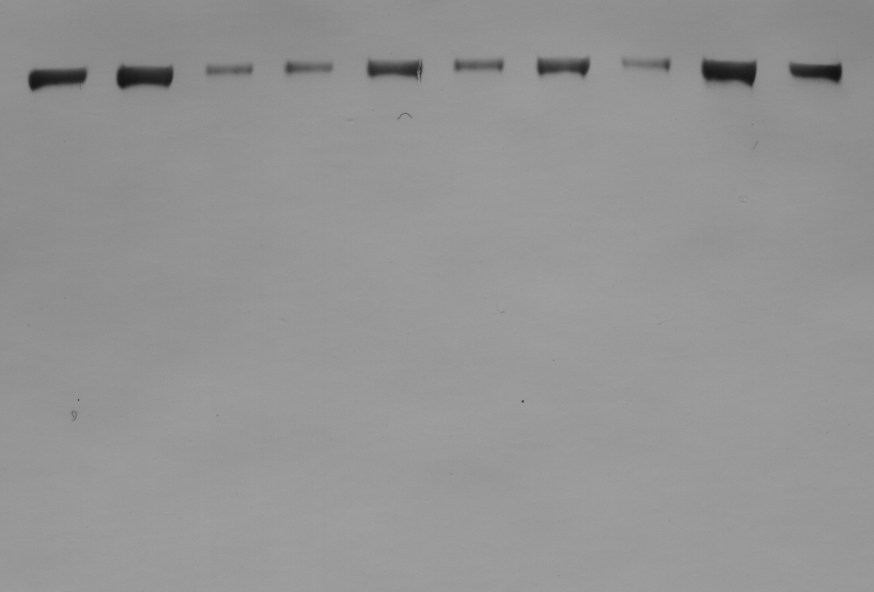

Supplement: S2 Fig — (ZIP) [file pone.0186993.s002.zip › Fig.8-Sirt1-PLoSONE-Original.tif]

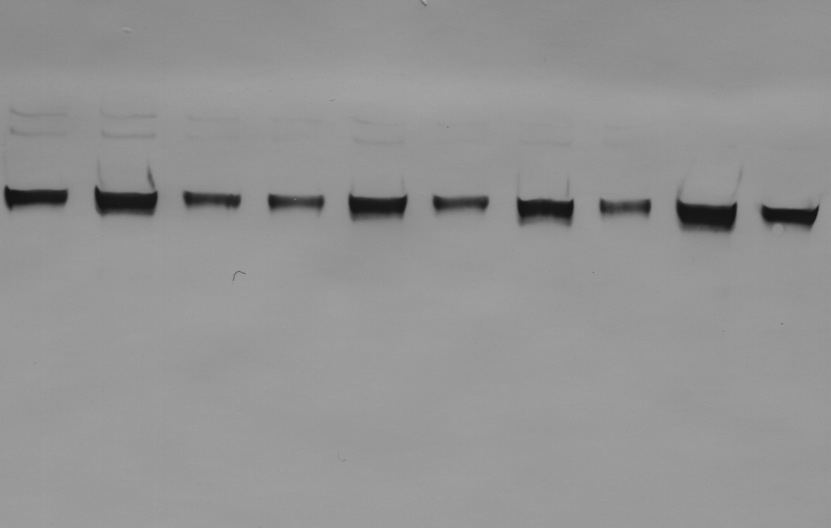

Supplement: S2 Fig — (ZIP) [file pone.0186993.s002.zip › Fig.8-Sox9-PLoSONE-Original.tif]

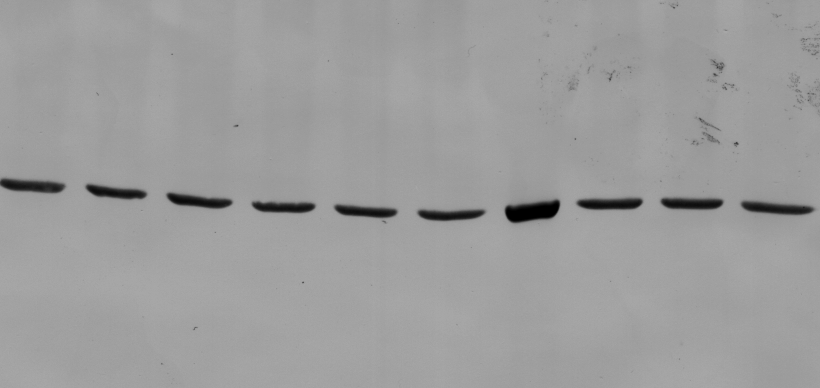

Supplement: S2 Fig — (ZIP) [file pone.0186993.s002.zip › Fig.8-ß-Actin1-PLoSONE-Original.tif]

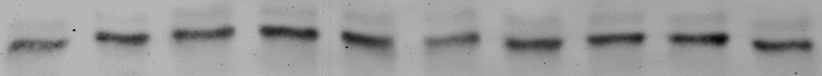

Supplement: S2 Fig — (ZIP) [file pone.0186993.s002.zip › Fig.8-ß-Actin2-PLoSONE-Original.tif]

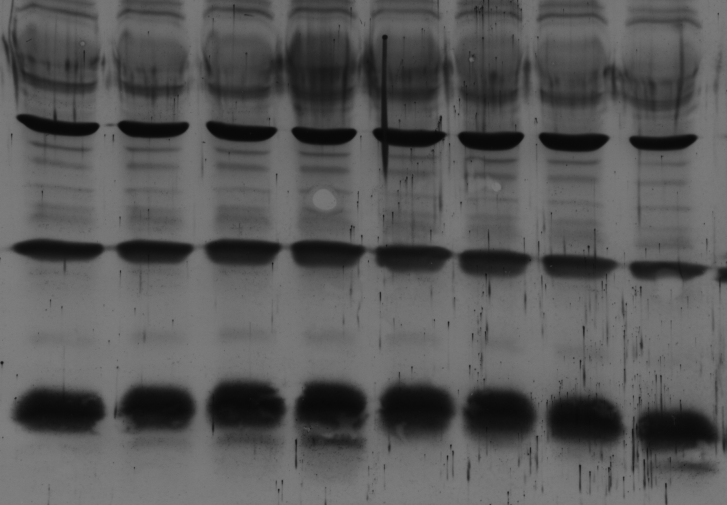

Supplement: S3 Fig — (ZIP) [file pone.0186993.s003.zip › Fig.9-Caspase-3-Actin-PLoSONE-Original.tif]

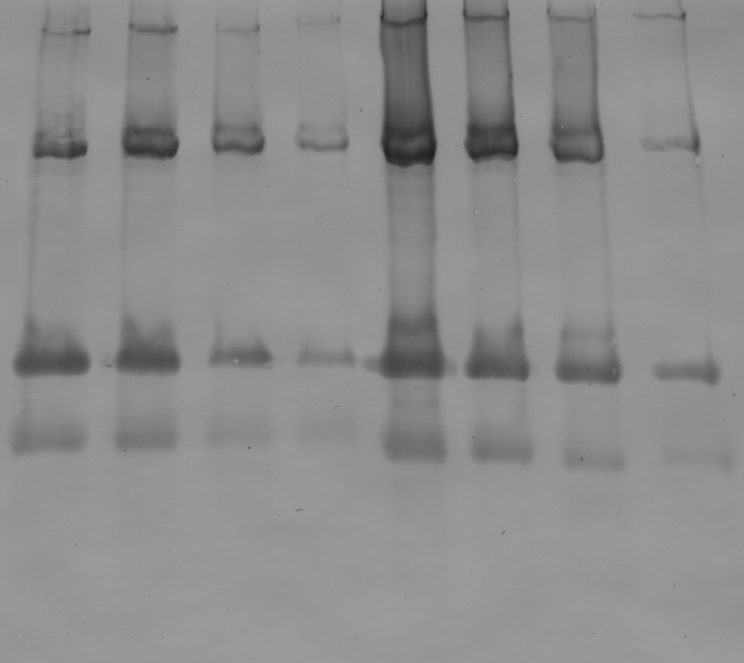

Supplement: S3 Fig — (ZIP) [file pone.0186993.s003.zip › Fig.9-Collagen II-PLoSONE-Original.tif]

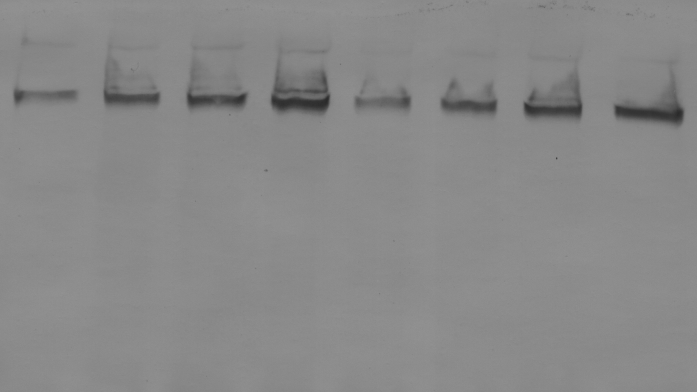

Supplement: S3 Fig — (ZIP) [file pone.0186993.s003.zip › Fig.9-COX-2-PLoSONE-Original.tif]

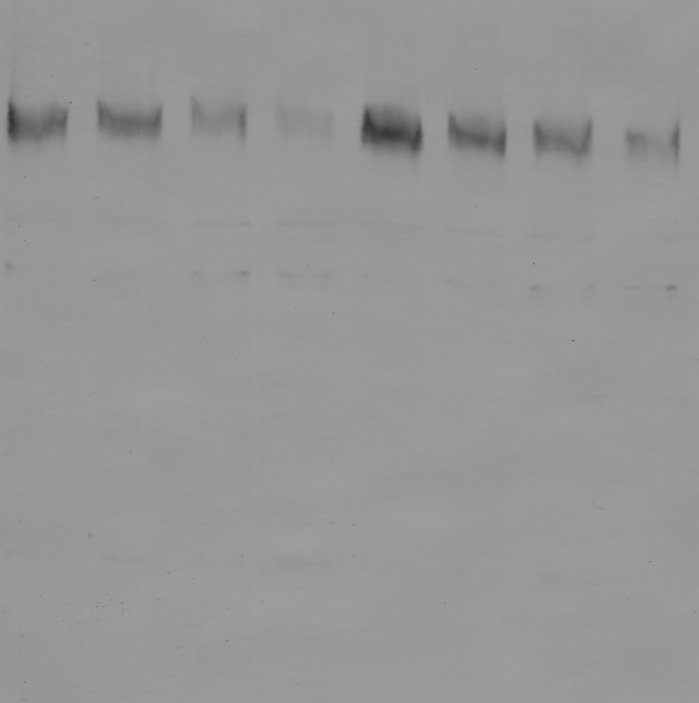

Supplement: S3 Fig — (ZIP) [file pone.0186993.s003.zip › Fig.9-Ki67-PLoSONE-Original.tif]

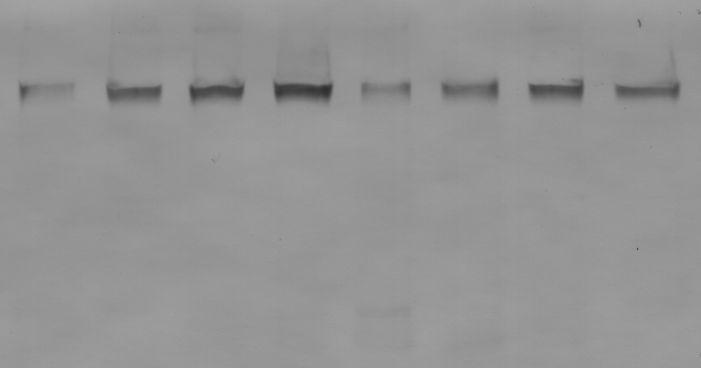

Supplement: S3 Fig — (ZIP) [file pone.0186993.s003.zip › Fig.9-MMP-13-PLoSONE-Original.tif]

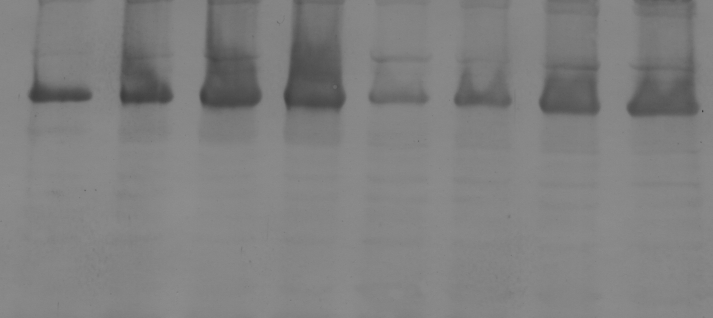

Supplement: S3 Fig — (ZIP) [file pone.0186993.s003.zip › Fig.9-MMP-9-PLoSONE-Original.tif]

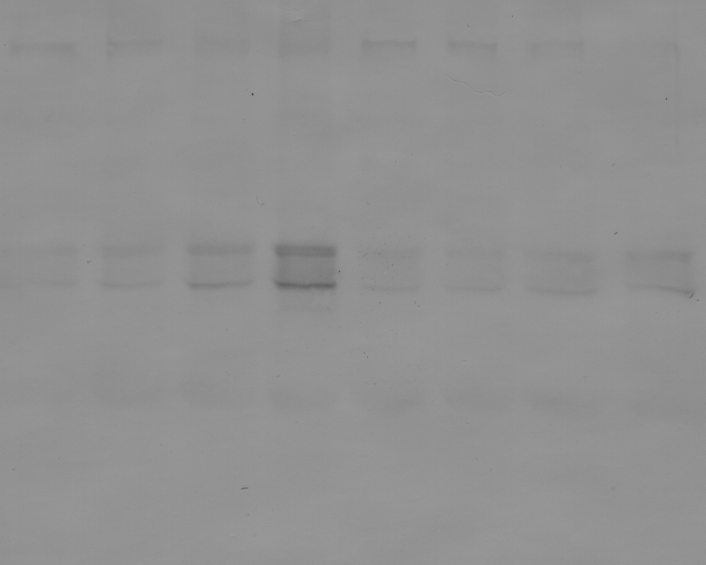

Supplement: S3 Fig — (ZIP) [file pone.0186993.s003.zip › Fig.9-NF-kB-PLoSONE-Original.tif]

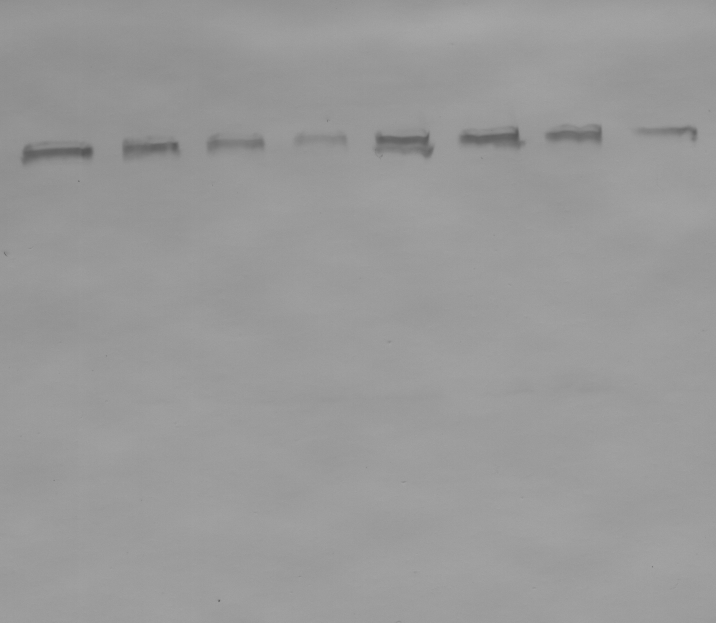

Supplement: S3 Fig — (ZIP) [file pone.0186993.s003.zip › Fig.9-Sirt1-PLoSONE-Original.tif]

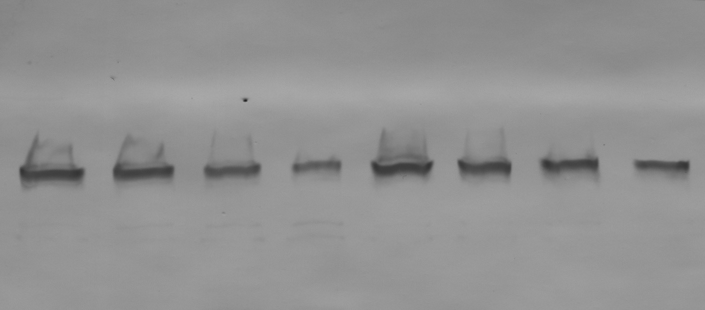

Supplement: S3 Fig — (ZIP) [file pone.0186993.s003.zip › Fig.9-Sox9-PLoSONE-Original.tif]

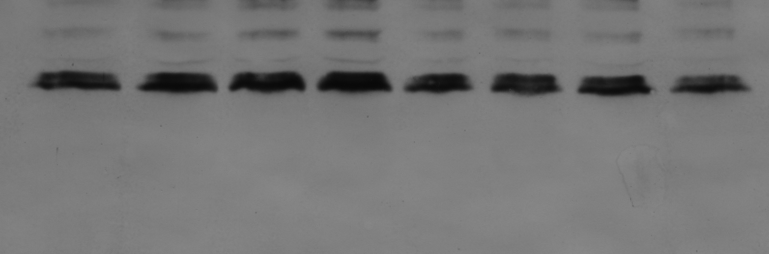

Supplement: S3 Fig — (ZIP) [file pone.0186993.s003.zip › Fig.9-ß-Actin1-PLoSONE-Original.tif]

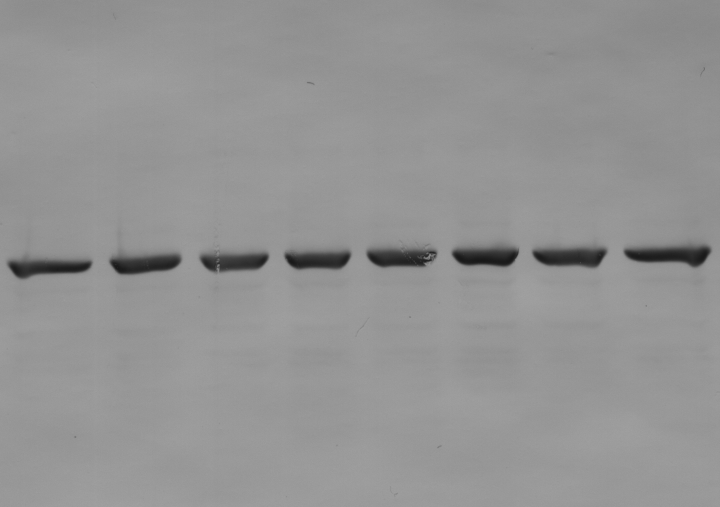

Supplement: S3 Fig — (ZIP) [file pone.0186993.s003.zip › Fig.9-ß-Actin2-PLoSONE-Original.tif]
